# Enhanced eicosapentaenoic acid production by a new deep-sea marine bacterium *Shewanella electrodiphila* MAR441^T^

**DOI:** 10.1371/journal.pone.0188081

**Published:** 2017-11-27

**Authors:** Jinwei Zhang, J. Grant Burgess

**Affiliations:** 1 Institute of Biomedical and Clinical Sciences, University of Exeter Medical School, Hatherly Laboratory, Exeter, United Kingdom; 2 School of Natural and Environmental Sciences, Newcastle University, Newcastle upon Tyne, United Kingdom; Osaka University Graduate School of Medicine, JAPAN

## Abstract

Omega-3 fatty acids are products of secondary metabolism, essential for growth and important for human health. Although there are numerous reports of bacterial production of omega-3 fatty acids, less information is available on the biotechnological production of these compounds from bacteria. The production of eicosapentaenoic acid (EPA, 20:5ω3) by a new species of marine bacteria *Shewanella electrodiphila* MAR441^T^ was investigated under different fermentation conditions. This strain produced a high percentage (up to 26%) of total fatty acids and high yields (mg / g of biomass) of EPA at or below the optimal growth temperature. At higher growth temperatures these values decreased greatly. The amount of EPA produced was affected by the carbon source, which also influenced fatty acid composition. This strain required Na^+^ for growth and EPA synthesis and cells harvested at late exponential or early stationary phase had a higher EPA content. Both the highest amounts (20 mg g^-1^) and highest percent EPA content (18%) occurred with growth on L-proline and (NH_4_)_2_SO_4_. The addition of cerulenin further enhanced EPA production to 30 mg g^-1^. Chemical mutagenesis using NTG allowed the isolation of mutants with improved levels of EPA content (from 9.7 to 15.8 mg g^-1^) when grown at 15°C. Thus, the yields of EPA could be substantially enhanced without the need for recombinant DNA technology, often a commercial requirement for food supplement manufacture.

## Introduction

Omega-3 polyunsaturated fatty acids (PUFAs), such as eicosapentaenoic acid (EPA, 20:5ω3) and docosahexaenoic acid (DHA, 22:6ω3), are important to human health. Nutritional insufficiencies of omega-3 PUFAs may have adverse effects on brain development and neurodevelopmental outcomes [1]. Omega-3 PUFAs are considered as therapeutic options which may reduce secondary neuronal damage initiated by traumatic brain injury [2]. They also have a protective role in age-related macular degeneration, and can prevent the harmful effects of chronic stress [3], and may contribute to the prevention of cognitive decline [4]. In addition, benefits for bone health and turnover have been reported [5]. Omega-3 fatty acids also play an important role in the modulation and prevention of heart failure and cardiovascular disease [6], and reduce the risk of development or progression of Alzheimer's disease [7].

Currently, the most prominent dietary sources of EPA and DHA are fish oil, supplies of which are unable to meet increasing global demand. In addition, fish may contain mercury, polychlorinated biphenyls, and other contaminants that have adverse effects on humans, particularly the developing foetus [8]. Furthermore, there are other potential problems associated with fish oils as a source of PUFA, such as: taste, odor, chemical stability as well as coextracted contaminants [9]. Nevertheless, the main problem of fish oil as a source of omega-3 fatty acids is its sustainability due to the worldwide decline of fish stocks [10]. Alternatively, natural production of omega-3 fatty acids by marine microbes is a potential alternative source, which would also be suitable for vegetarians. Furthermore, most bacteria can be grown on waste nutrients from other agricultural or industrial processes. Therefore, dietary sources of EPA and DHA from microbial biomass appear to be particularly promising and an area in which further research is warranted.

So far, bacterially derived PUFAs were reported mainly from Gram-negative bacteria [11], from two bacterial phyla: the *Gammaproteobacteria* (e.g., *Shewanella*, *Moritella*, *Colwellia*, *Alteromonas*, and *Photobacterium*) and the *Bacteroidetes* (e.g. *Flexibacter* and *Psychroserpens*) [12]. These EPA/DHA producing bacteria include psychrophiles and piezophiles, and were isolated from polar regions and the deep sea [13–19], as well as mesophiles isolated from a temperate estuary [20] and from shallow seawater samples [2,21–23]. However, it is unclear why these bacteria produce omega-3 fatty acids. Production of EPA by some bacteria increases as temperature decreases, leading to the hypothesis that these molecules may be important for growth at low temperatures [11,14,24]. Cells must cope with decreases in temperature by modulating the lipid composition of their membrane, which can crystallize or enter nonbilayer phases at low temperatures [25]. EPA was not required for low-temperature growth in the deep-sea bacterium *Photobacterium profundum* [26], but it may be required for low temperature growth in *Shewanella* [11,18,27]. Bacteria adjust their membrane lipid composition by modifying certain types of fatty acids to maintain membrane viscosity in response to environmental changes, such as temperature, pressure or salt concentration.

[28]. A number of studies are available on the physiology of bacterial PUFA production under varying culture conditions [13,28,29]. These studies indicate that PUFA biosynthesis could be manipulated by changing environmental conditions. Metabolic engineering of bacterial PUFA gene clusters into *E*. *coli* as a host, has also been extensively studied for the production of PUFA, however, the yield of EPA/DHA reported was low (e.g. 2.2mg/g EPA and 4.1% TFA) [4,30]. Thus, continued research utilizing a variety of bacterial strains is warranted to more fundamentally understand PUFA biosynthesis and to continue to explore non-recombinant natural sources of microbially produced omega-3 fatty acids.

We recently identified a psychrophilic strain *Shewanella electrodiphila* MAR441^T^ that was isolated from sediment from a Mid-Atlantic Ridge (MAR) non-vent site at a depth of 2,500 m. This species was capable of producing relatively high yields of EPA (up to 24 mg g^-1^) when grow at all growth temperatures from 4 to 25°C [31]. In the current study, we have extended this work by significantly improving production of PUFA by varying the carbon source and using artificial sea water with different concentrations of Na^+^, time of culture and temperature. In addition, cerulenin treatment and chemical mutation by N-methyl-N′-nitro-N-nitrosoguanidine (NTG) were used in order to enhance secondary metabolite production of PUFAs. The acyl chain speciation of the major phospholipid classes and non-esterified fatty acid (NEFA) fraction produced by strain MAR441^T^ are also described.

## Methods

### Cultivation conditions for biomass

For biomass production, strains were inoculated into 10 ml of Zobell’s broth (ZB) [32], and incubated at 15°C until turbidity was apparent by optical density (OD) 600 nM. The 10 ml cultures were then used to inoculate 90 ml volumes of marine broth (MB) contained in 500 ml conical flasks pre-rinsed with chloroform. Flasks were incubated at 15°C (or indicated temperatures) with agitation provided by a magnetic stirrer or orbital shaker (180 rpm) for 24–48 h until sufficient mass of estimated late-log phase cells were present for harvest. Cell mass from broth cultures was collected by centrifugation at 4500 g for 20 min. Cell pellets were resuspended in 100 ml M9 solution (22mM KH_2_PO_4_, 22mM Na_2_HPO_4_, 85mM NaCl, 1mM MgSO_4_) and recentrifuged followed by rinsing with 0.1% ammonium acetate and frozen. The washed cell pellets were suspended in 2.0 ml saline and lyophilised in pre-weighed containers prior to lipid extraction as descripted previously [31]. The collected samples were then stored at -20°C followed by -80°C, for fatty acids analysis.

### The effect of temperature

The temperature-growth response (4–30°C) of strain MAR441^T^ was studied by growing in marine broth (MB). Growth was observed and samples collected every day for five days, centrifuged and washed with sterile M9 solution. The generation time was used to evaluate the cell growth in the exponential phase according to previous methods [33].

### Effect of carbon and nitrogen source

Strain MAR441^T^ was grown on various sole carbon sources (L-alanine, L-leucine, L-proline, L-serine, propionic acid, glucose, glycerol, Tween 80, 60 and 40) and nitrogen sources (Urea and (NH_4_)_2_SO_4_) in triplicate or duplicate (see Supplementary S2 Table). Negative Control (NC) medium contained 0.01% (w/v) yeast extract in seawater. Modified media for sole carbon/nitrogen source cultures was 0.5% (w/v) carbon/nitrogen source and 0.01% (w/v) yeast extract in 0.22 µm filtered and sterilized natural seawater [34]. Optimal Medium (OM) contains 0.5% (w/v) Proline and 0.5% (w/v) (NH_4_)_2_SO_4_) in NC; Cultures were incubated at 15°C, in triplicate in 50-ml ZB contained within 200-ml flasks pre-rinsed in chloroform with shaking (180 rpm) for 36–48 h until sufficient mass of estimated late-log phase cells was present for harvest.

### Cultivation conditions with treatment of cerulenin

Ten ml seed cultures of MAR441^T^ were used to inoculate 90 ml volumes of marine broth contained in 500 ml conical flasks, where the antibiotic cerulenin (Merck), in 50% (v/v) ethanol (1 mg ml^-1^) was added at various concentrations (0, 0.5, 1, 2.5, 5 and 7.5 µg ml^-1^) prior to cultivation. Flasks were incubated at 4 and 15°C respectively. The growth of cells was monitored turbidometrically at 600 nm.

### Induction of mutations with nitrosoguanidine (NTG)

N-methyl-N’-nitro-N-nitrosoguanidine (NTG) purchased from Tokyo Kasei Kogyo Co., Ltd. Japan, was used as chemical mutagen. Chemical mutagenesis was carried out according to the literature (Kotchoni et al. 2003; Liu et al. 2004). Exponentially growing cells of strain MAR441^T^ were harvested from 3 ml of fermentation broth by centrifuging at 1600 g for 10 min at room temperature. The pellet was washed twice with 0.85% NaCl solution and then resuspended in 5 ml 0.1 M phosphate buffer, producing a suspension containing ~10^5^ cfu l^−1^ (colony forming units l broth^−1^). The cells were then exposed to two NTG concentrations (300 µg l^−1^ and 500 µg l^−1^) for 12 h at 4 and 15°C by adding appropriate volumes of NTG stock (720 µg l^−1^ NTG in 0.1 M phosphate buffer, pH 6.5) to the cell suspensions. 1 ml samples of the serially-diluted culture were then spread on agar plates for mutant discovery. The agar plates were incubated for 2 days at 4 and 15°C respectively, and the resulting colonies (more than 1000 colonies) were taken off the agar plates by random selection based on morphology (e.g. size and colour), 50 colonies were further chosen for evaluation of their ability to produce PUFAs. The transformation experiments involving the selected mutants were conducted using the same culture methods used for the parent strain.

### Scanning electron microscopy

Cells were processed for scanning electron microscopy (SEM) (Cambridge Stereoscan 240). The samples were fixed in 2% glutaraldehyde in 0.2 M Sorenson’s phosphate buffer (pH 6.8) for 12 h, then rinsed in Sorenson’s phosphate buffer twice for 15 mins and dried in an alcohol series up to 100%. The samples were then CO_2_-critical point dried by a Samdri 780 Critical Point Dryer, mounted on an aluminium stub with Achesons Silver ElectroDag and coated with 15 nm gold/palladium (40/60) using a polaron SEM coating unit and then observed using SEM.

### Lipid extraction, preparation of fatty acid methyl esters and analysis

Cell samples that prepared as mentioned above in “Cultivation conditions for biomass were harvested by centrifugation (4500 g, 4°C) and frozen at -20°C followed by -80°C, before freeze-drying. Freeze dried biomass was accurately weighed; an internal standard (2-Terthiophene triheneicosanoin, n-21:0, Sigma) was added. Fatty-acyl methyl esters were prepared by using sulfuric-acid-catalysed trans-esterification [35,36]. After the transmethylation, fatty acid methyl esters (FAMEs) were extracted with n-hexane, concentrated under a stream of oxygen-free dry nitrogen at 37°C, to give a total lipid extract (TLE). Fractionation of phospholipids from the TLE was accomplished by thin-layer chromatography (TLC). Portions of sample TLEs were applied to silica gel plates (Silica gel 60 F254, Merck) that had been activated at 100°C for 1h. Plates were developed in CHCl_3_/MeOH/CH_3_COOH/H_2_O (85:15:10:3.5, v/v/v/v). Samples were visualised by iodine vapour and identified by comparison with known standards which were identified with rhodamine-6-G, ninhydrin and Dragendorff stains [37]. The lipid classes were separated by silica gel (1:30 w/w of lipid) column chromatography by successive elution with chloroform (1:10 m/v of lipid), acetone-methanol (9:1 v/v;1:15 w/v of lipids) and methanol (1:10 w/v of lipid) to get neutral-(NL), glyco-(GL) and phospho-(PL) lipids respectively. All fractions along with total lipids were transmethylated using sodium methoxide (0.5 M) to obtain the FAMEs and analyzed with a modified method published previously [38] Analyses of the FAME preparations were performed with a Hewlett-Packard model 7890A GC (Varian CP-3800, Varian, Inc. 2700 Mitchell Drive Walnut Creek, CA 94598-1675/USA) equipped with type DB225 capillary column (BPX70, 10 m x 0.1 mm, 0.2 µm; J & W Scientific, Folsom, Ca, USA) with programmed temperature of 170°C–220°C, a linear increase at 5°C min^-1^, injection and detection temperature maintained at 250 and 260°C, respectively, and helium as the carrier gas. GC/MS analysis was carried out with Agilent 5975 GC/MS (Agilent Technologies Co., Ltd., Palo Alto, USA) equipped with HP-5ms Capillary GC-MS Column (Agilent 19091S-433, 30 m x 0.25 mm, 0.25 µm), temperature programme 120°C for 1 min, increased at 8°C min^-1^ to 260°C, which was maintained for 10 min with He as the carrier gas. MS operating conditions were as follows: electron multiplier, 2,000 V; transfer line, 250°C; electron impact energy, 70 eV; scan threshold, 50; 1.3 scans s21 with a mass range of 50 to 500 atomic mass units; and solvent delay, 2.35 min. Compounds were identified by comparison of their retention times with those of known standards, and sample mass spectra data were compared to the mass spectra data of 275, 000 compounds in the Wiley 275 spectra library.

## Results

### Fatty acid composition of phospholipid classes

The amount of total lipid was 12.5% of dry cell mass from 10°C MB cultures and the content of phospholipids and neutral lipids were about 72% and 28% of total lipid, respectively (**Table 1**). As identified by TLC from fractionation of TLEs, phosphatidyl ethanolamine (PE) was the dominant lipid class in phospholipids (50%) followed by phosphatidyl glycerol (PG) (40%). About 5% of diphosphoglyceride (DPG) and 3% of lysophosphatidylethanolamine (LPE) were also detected with some unidentified phospholipids (2%). The fatty acid compositions of TLEs and their derived PE, PG and DPG fractions are shown in **Table 2**. There was a near equal proportion of SCFAs within the PE and PG fractions. However, PE contained a higher proportion of BCFAs components (25% PE versus 15% PG) mainly due to a higher percentage of i-13:0 (9% PE versus 6% PG) and i-15:0 (15% PE versus 8% PG). In contrast, PG contained a greater proportion of MUFAs (28% versus 19%) which was due to slightly higher proportions of all monounsaturated acyl species. PUFA were present in both phospholipid classes, although PG contained a higher percentage of total PUFAs (17% versus 14%) and EPA (14% versus 10%). Free fatty acids (FFA) or non-esterified fatty acids (NEFA) were recovered from the TLC plates (**S1 Fig**), and found with high content of MUFAs and PUFAs, especially the high content of EPA and n-16:1ω7c (18.7% and 26.5%, respectively) and with less SCFAs and BCFAs. Whereas, DPG contained higher SCFAs and BCFAs, especially n-13:0 and i-15:0 (28.4% and 15.7%, respectively), and less MUFAs and PUFAs, from which 6.5% of EPA was also detected. Thus, a mass balance calculation of the percentage of EPA, based on a qualitative assessment of probable phospholipid class distribution did balance (**Table 2**).

**Table 1:** Lipid composition of strain MAR441^T^ when grown in marine broth at 10°C.^a^.

Biomass (g dry cell l^-1^)	2.66 ± 0.1
Total lipid (% of dry weight)	12.5 ± 0.25
Neutral lipids (% of total lipids)	28 ± 0.37
Free fatty acid (FFA) (% of total lipids)	15 ± 0.22
Phospholipids (PL) (% of total lipids)	72 ± 0.65
Phosphatidylethanolamine (PE) (% of PL)	50 ± 0.43
Phosphatidylglycerol (PG) (% of PL)	40 ± 0.21
Diphosphatidylglycerol (DPG) (% of PL)	5 ± 0.15
Lysophosphatidylethanolamine (LPE) (% of PL)	3 ± 0.1
Unidentified phospholipid	2 ± 0.1
EPA (% of total lipids) ^a^	13.56 ± 0.43
EPA (mg g^-1^)	16.95 ± 0.83
Lipid g l^-1^	0.3325 ± 0.1
Lipid (mg g^-1^)	125 ± 0.54

^a^ The values are means of three samples.

**Table 2:** Distribution of major fatty acid in total and different lipid classes in strain MAR441^T^ when grown in marine broth at 10°C.

Fatty acids	TFA	FFA	DPG	PE	PG
n-12:0	2.1	2.2	2.5	0.3	0.5
n-13:0	22.92	17.6	28.4	24.8	19.5
n-14:0	4.11	2.85	3.1	3.7	4.2
n-15:0	2.25	1.3	2.8	1.8	2.7
n-16:0	10.99	5.5	9.2	10.5	12.2
n-17:0	0.56	0.1	0.5	0.7	0.5
n-18:0	0.44	0.1	0.5	0.4	0.3
**Σ SCFA**	**43.37**	**29.65**	**47**	**42.2**	**39.9**
i-13:0	7.23	3.9	9.4	8.9	5.7
i-14:0	0.33	0.67	0.2	0.6	0.3
ai-15:0	0.68	0.35	1.7	0.6	0.46
i-15:0	10.29	7.5	15.7	14.5	7.6
i-17:0	0.2	0.1	0.15	0.4	0.5
**Σ BCFA**	**18.73**	**12.52**	**27.15**	**25.0**	**14.6**
n-15:1ω6	0.09	0.88	0.2	1.2	1.3
n-16:1ω7	13.65	26.5	12.5	12.5	19.4
n-17:1ω8	0.28	0.3	0.3	0.1	0.5
n-18:1ω9c	0.53	0.42	0.2	0.45	0.7
n-18:1ω7c	4.88	4.24	3.5	4.5	6.1
n-20:1ω9	0.19	0.3	0.1	0.2	0.3
**Σ MUFA**	**19.62**	**32.64**	**16.8**	**19.0**	**28.3**
n-18:2ω6t	1.16	2.5	0.7	1.2	0.8
n-18:3ω6t	0.09	0.2	0.05	-	0.1
n-18:3ω3	0.11	0.5	0.8	0.2	0.1
n-18:4ω3	0.31	0.4	0.1	0.6	0.3
n-20:2	0.08	0.1	-	0.1	0.1
n-20:3ω6	0.04	0.1	-	-	-
n-20:4ω6	0.2	0.2	0.1	0.2	0.1
n-20:3ω3	0.06	0.2	-	0.1	0.1
n-20:4ω3	0.59	0.8	-	1.1	0.6
**n-20:5ω3**	**15.01**	**18.7**	**6.5**	**9.5**	**14.2**
n-22:2ω6	0.04	0.2	0.1	0.1	-
n-22:4ω6	0.04	0.1	-	-	-
n-22:5ω3	0.49	0.5	0.2	0.35	0.5
**Σ PUFA**	**18.22**	**24.5**	**8.55**	**13.45**	**16.9**
Others	0.06	0.69	0.5	0.45	0.34
Total	100	100	100	100	100
ACL^a^	15.62	16.02	14.83	15.27	15.79

^a^ACL, average chain length

Values are means of three samples; SCFA, straight chain fatty acids; BCFA, branched chain fatty acids; MUFA, monounsaturated fatty acids; PUFA, polyunsaturated fatty acids; TFA, total fatty acids; EPA, eicosapentaenoic acid (20:5ω3); and (–), not detectable.

**Fig 1:**
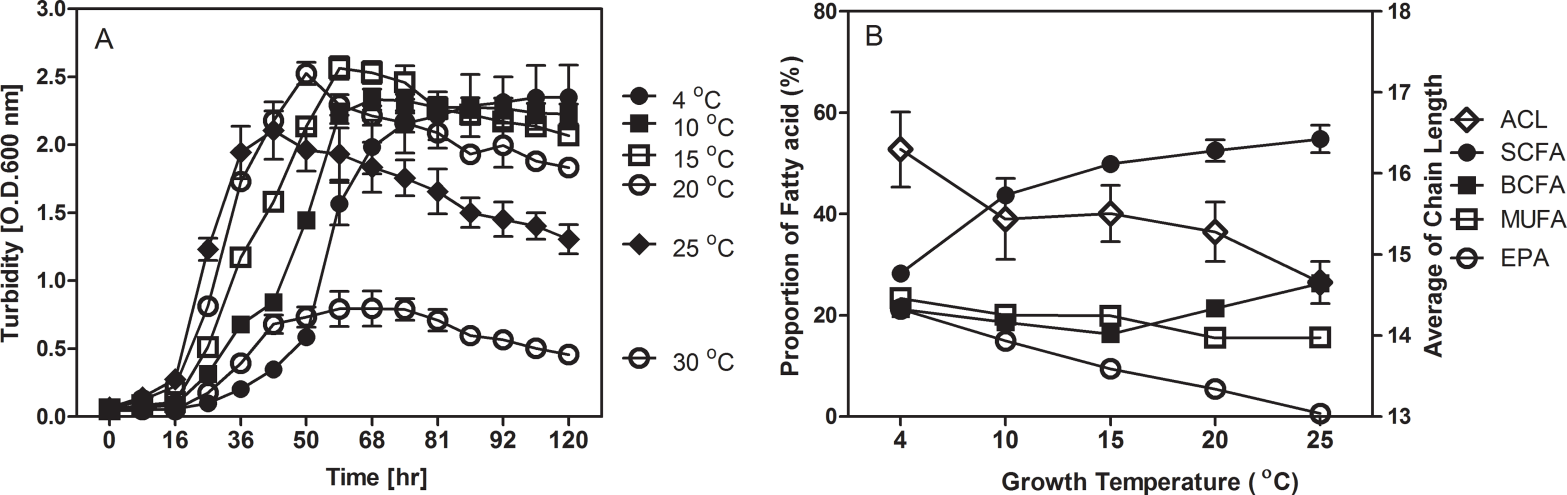
**(A)** Growth curves of strain MAR441^T^ with shaking at various temperatures under atmospheric pressure in marine broth medium; **(B)** Change in average chain length (ACL, open diamonds) and relative proportion of whole cell FAs in strain MAR441^T^ grown at 4, 10, 15, 20 and 25°C in marine broth medium. Straight chain fatty acids (SCFAs, filled circles), branched chain fatty acids (BCFAs, filled boxes), monounsaturated fatty acids (MUFAs, open boxes); eicosapentaenoic acid (EPA, open circles). The experiments were carried out in triplicate and values are means of three samples based on our recent published data (Supplementary S1 Table -).

The effect of growth temperature on the percentage composition of individual fatty acids in MAR441^T^ grown between 4 and 25°C is as described in our recent publication (Supplementary S1 Table [39]) and further analysed as **Fig 1B**. Growth at temperatures 4°C and 10°C which are below the optimal growth temperature range of 15–20°C, resulted in a higher percentage of EPA and n-16:1ω7c, and a lower percentage of n-13:0 and n-16:0 compared to growth at 15°C or above 20°C, the optimum growth temperature range. Growth within the optimal region achieved the highest percentage of n-15:0 and n-16:0, while iso-tridecanoic acid (i-13:0) and the sum of monounsaturated fatty acids were at their lowest levels. At growth temperatures above the optimal region, the percentage of iso-pentadecanoic acid (i-15:0), n-17:0 and n-17:1ω8 were maximal, while the percentage of n-13:0 increased with increasing growth temperature. With increasing temperature from 4 ºC to 25 ºC, the proportion of monounsaturated fatty acids (MUFAs) and polyunsaturated fatty acids (PUFAs) decreased, with an increase in the proportion of straight chain fatty acids (SCFAs), whereas branched chain fatty acids (BCFAs) were at their lowest concentrations at 15°C. The values of average chain length (ACL, calculated after reference [40]), are 16.29–14.67 and quantitative levels of EPA decreased with increasing growth temperature (24–0.2 mg g^-1^ cells dry weight) at all growth temperatures from 4–25°C (**Fig 1B**).

### Time course of cell growth and FA production

**Fig 2:**
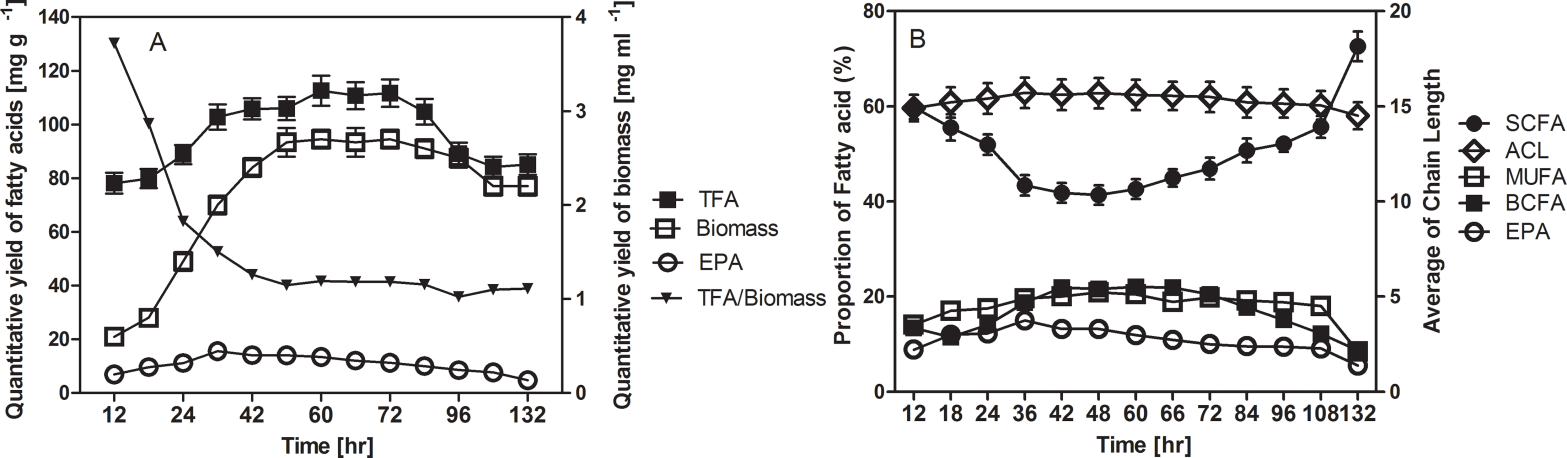
**(A)** Time period of cell growth (filled circles) and quantitative yield of whole cell TFAs and EPA in strain MAR441^T^ grown in marine broth medium at 15°C during the time period. Total fatty acids (TFAs, filled boxes); eicosapentaenoic acid (EPA, open circles); quantitative yield of biomass (open boxes). **(B)** Change in average chain length (ACL, open diamonds) and relative proportion of whole cell FAs in strain MAR441^T^ grown in marine broth medium at 15°C during the time period. Straight chain fatty acids (SCFAs, filled circles), branched chain fatty acids (BCFAs, filled boxes), monounsaturated fatty acids (MUFAs, open boxes); eicosapentaenoic acid (EPA, open circles). The experiments were carried out in triplicate and values are means of three samples based on **S1 Table**.

Fatty acids are intracellular products in strain MAR441^T^ which shows a typical time course of EPA production at 15°C (**Fig 2 and S1 Table**). The increase in lipid and EPA content parallels that of cell growth (**Fig 2A**). After 36 h of cultivation, the cells entered the late exponential or early stationary phase and the content of TFAs, PUFAs and EPA reached their maximum of 103.1 mg g^-1^, 17.7 mg g^-1^and 15.48 mg g^-1^ respectively, when EPA is at its highest content of 15% of TFA. In this experiment a 5% culture inoculum was used, and the maximum TFA concentration reached 113 mg g^-1^ after 60 h culture, with ca. 16.8 mg g^-1^ of PUFA and 13.4 mg g^-1^ of EPA. The TFAs and EPA were produced with a stable quantitative yield of 90–120 mg g^-1^ and 11–15 mg g^-1^ for 60 hours from 24 h to 84 h time period culture, during which the percentage composition of SCFAs, BCFAs, MUFAs, PUFAs and EPA was kept at a relative stable levels of 41–46%, 19–22%, 18–20%, 13–17% and 10–15% respectively, and the average chain length remained more or less constant from 15.21–15.73. However, an increase in cell growth during the exponential phase led to a decrease in SCFAs from a high level of 60–43% in favour of BCFAs, MUFAs and PUFAs, due to the great decrease of n-13:0 with a corresponding increase of n-16:0, i-C15:0, n-16:1ω7 and EPA, which contributing the increase of average chain length. Interestingly, the cell death during the late stationary phase and death phase of cell culture led to a great increase in SCFAs from 50–73% at the expense of BCFAs, MUFAs and PUFAs, due to a surprising rise of n-13:0 from 29–63%, which was accompanied by a decrease of n-16:0, i-15:0, n-16:1ω7 and EPA, as well as the average chain length.

### Effect of Na^+^ on cell growth and PUFA production

**Fig 3:**
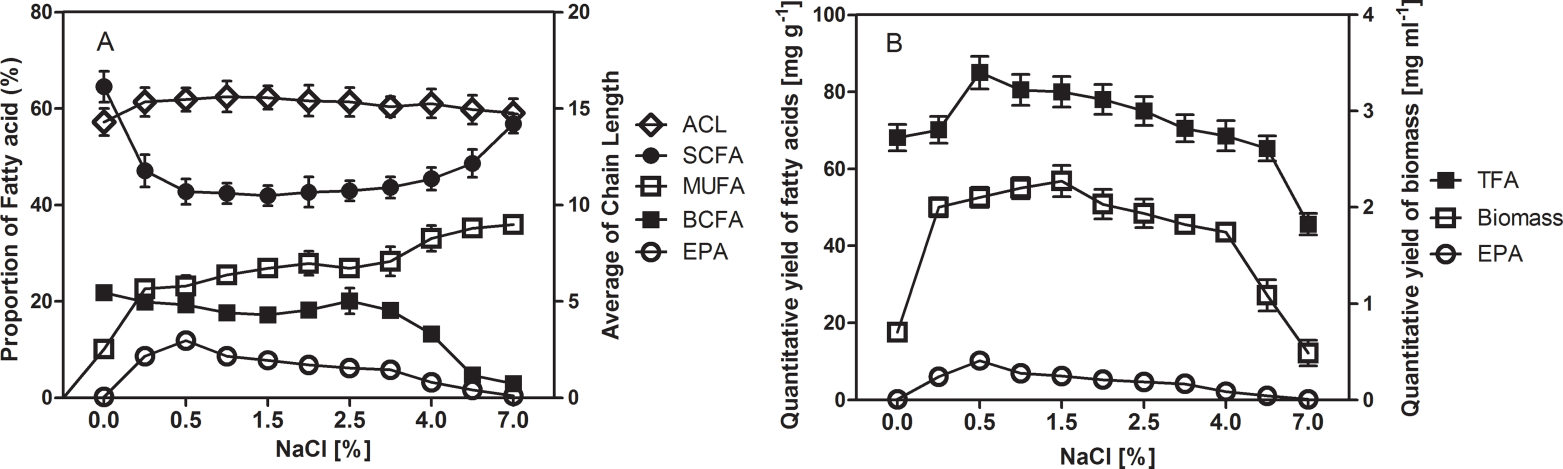
**(A)** Change in average chain length (ACL, open diamonds) and relative proportion of whole cell FAs in strain MAR441^T^ grown in ZB medium at 15°C with various concentration of NaCl. Straight chain fatty acids (SCFAs, filled circles), branched chain fatty acids (BCFAs, filled boxes), monounsaturated fatty acids (MUFAs, open boxes); eicosapentaenoic acid (EPA, open circles). **(B)** Quantitative yield of biomass (open boxes), TFA and EPA in strain MAR441^T^ grown in ZB medium at 15°C with various concentration of NaCl. Total fatty acids (TFAs, filled boxes); eicosapentaenoic acid (EPA, open circles). The experiments were carried out in triplicate and values are means of three samples based on **S2 Table**.

This bacterium required Na^+^ for growth; growth occurred at Na^+^ concentrations ranging from 0.051 M (0.3%) to 1.197 M (7%) with an optimum between 0.051 and 0.513 M (3%), which is consistent with the sea-water environment containing about 19.45 g l^-1^ (0.85 M) Na^+^; the growth was inhibited at a Na^+^ concentration less of 0.3% or above 7%. Different concentrations of sodium chloride were supplemented to the regular no salt contained ZB medium to study their effects in biomass formation, fatty acid biosynthesis and desaturation reactions were investigated (**Fig 3 and S2 Table**). A significant difference in biomass formation was observed between cultures within optimum Na^+^ concentrations and cultures without Na^+^ or with high Na^+^ concentrations. 1.7–2.2 g l^-1^ biomass and 2–10 mg g^-1^ EPA were achieved when the Na^+^ in the ZB medium were controlled between 0.051–0.684 M, while only 0.4–1.0 g l^-1^ biomass and 0–0.18 mg g^-1^ EPA were produced when the medium were supplemented without or with higher concentration of Na^+^ (>0.85 M). A low percentage composition (0.2%) of EPA was produced by MAR441^T^ cells, with high levels of SCFAs (64.5%), when the culture medium contained no metal ions. When the medium was supplemented with a low concentration of Na^+^ (0.3%), a decrease of SCFAs in favour of BCFAs, MUFAs and PUFAs was observed, the decrease of n-13:0 corresponded to the increase of n-14:0, n-16:0, i-13:0, n-16:1ω7, n-18:1ω7 and EPA, contributing to the increase of average chain length from 14.27 to 15.32. With 0.3% Na^+^ there was a significant increase in the productivity of EPA from 0.4% to 8.6%. EPA production reached its maximum at 15.4% of TFAs when the concentration of Na^+^ was maintained at 0.5%. However, increasing Na^+^, led to a decrease in the proportion of EPA from 15.4% to 0.4% when 7% of Na^+^ was added. Nevertheless, at 0.5–3% Na^+^, the percentage composition of SCFAs, BCFAs, MUFAs and PUFAs in MAR441^T^ cells was stable. Whereas, growth on 4–7% Na^+^ increased the proportion of SCFAs, which was accompanied by a decrease in the percentages of BCFAs, MUFAs and PUFAs, and this was mirrored by a corresponding decrease of average chain length.

### Effect of sole carbon and nitrogen source on FA production

To elucidate global adaptation mechanisms of membrane fatty acids in strain MAR441^T^ in response to growth at a low temperature on several different carbon sources, the degree of saturation (i.e. saturated/unsaturated ratio), the degree of polyunsaturation (i.e. PUFA/MUFA ratio) and average chain length (ACL) of fatty acids were determined and compared. The changes in fatty acid composition of MAR441^T^ grown on various sole carbon/nitrogen sources at 15°C are shown in **S3 Table**.

Growth on L-leucine, L-alanine, L-serine, L-proline, glucose, glycerol, pyruvate, Tween 80, Tween 60, Tween 40, urea and (NH_4_)_2_SO_4_ produced a fatty acid composition similar to that obtained in complex media MB or ZB. Only some differences in percentage composition in quantitative levels of TFA were apparent.

Growth on complex media MB or ZB achieved high production of n-16:1ω7 (14%) and EPA (13–15%). However, growth on MB lowered production of SCFAs, such as n-13:0 (23% versus 36%), which was mirrored by a corresponding increase in BCFAs due to the higher amount of i-13:0 (7% versus 3% for ZB) and i-15:0 (10% versus 3%), as well as by a higher quantitative yield of EPA (15.5 mg g^-1^ versus 14.2 mg g^-1^) but with similar levels of ACL (15.57 versus 15.49). PUFA levels produced by strain MAR441^T^ in MB and ZB (0.91 and 1.11 of polyunsaturation degree) were substantially higher compared with other organic substrates, where almost equal quantities of PUFA have been observed (0.1–0.86 of polyunsaturation degree).

Growth on L-alanine and L-serine produced a very similar fatty acid percentage composition as well as ACL (15.39% versus 15.38%), with a very low proportion of n-13:0 (24–26% versus 32–51% for other carbon sources excluding glycerol). This was accompanied by a rise in the percentages of other fatty acids with different levels, especially on n-16:1ω7 (11–15%) and EPA (11–13%). However, growth on L-alanine led to a higher quantitative yield of EPA (12.57 mg g^-1^ versus 11.13 mg g^-1^).

Growth on L-leucine resulted in the highest production of n-13:0 (45.7% versus 21–41% for other carbon sources) and i-15:0 (6.5%) at the expense of MUFAs and PUFAs, such as n-16:1ω7, n-18:1ω7c and EPA. This was mirrored by a corresponding decrease in the quantitative yield EPA (5.68 mg g^-1^) and ACL of 14.73.

Growth on L-proline decreased the proportion of BCFAs, such as i-13:0 (4% versus 5–9% for other carbon/nitogen sources excluding glycerol and Tween medium). This was accompanied by a rise in the percentages of n-18:1ω7 (7.1% versus 2–6% excluding Tween 60) and EPA (15.6% versus 4–12%). Furthermore, the increased proportion of EPA was mirrored by a corresponding increase in the quantitative yield (15.23 mg g^-1^ versus 4–11 mg g^-1^ for other carbon/nitrogen sources), which was comparable to MB cultures (15.5 mg g^-1^), and the ACL was in higher value (15.58).

The fatty acid composition of glucose cultured cells was very similar to that of ZB cultured cells. The only difference was that the former had a higher content of BCFAs (12.3% versus 7.7% for ZB) and lower proportion of PUFAs (12% versus 19%).

Growth on glycerol led to lower SCFA and BCFA content due to the lowest proportion of n-13:0 (21% versus 24–51% for other carbon/nitrogen sources) and i-13:0 (3.4% versus 4–9%, excluding Tween medium), whereas n-16:1ω7 at its highest point (29% versus 6–15%) which contributed a high level of MUFAs (34.7% versus 12–27%), in TFAs (saturation degree of 0.92), and percentage of EPA production was in a reasonable level (10%) as well as the quantitative yield (10.66 mg g^-1^).

Growth on Tween 80, 60 and 40 exhibited a marked alteration in fatty acid composition and the quantitative yield of EPA (4–7 mg g^-1^ versus 10–12 mg g^-1^ for other carbon sources) and cells (1.5–1.7 g l^-1^ versus 1.8–2.3 g l^-1^). The cultivation of strain MAR441 cells with Tween 80 (35% content of n-18:1ω9c oleic acid) caused the increase of MUFAs up to 48% in cellular lipids (saturation degree of 0.7) while inhibiting the production of PUFAs (polyunsaturation degree of 0.113). Growth on Tween 60 led to 10% of n-16:0 and 33% of n-18:0 causing the increase of SCFAs up to 77% in TFAs (saturation degree of 4.0) and therefore inhibited the BCFAs, MUFAs and PUFAs production. Growth on Tween 40 resulted in a high content of n-18:0 (15%) and n-16:1ω7c (25%), causing an increase of MUFAs up to 32% in TFAs, the degree of saturation and polyunsaturation were 1.2 and 0.29 respectively. However, the average chain length from these tween media were among the highest levels, 15.79–16.16 versus 14.73–15.62 for other carbon sources.

The percentage of EPA from the L-leucine, L-serine and L-glucose cultures was comparable to that obtained when grown in ZB at 15°C (10.2% versus 9–11%). These higher proportions of EPA corresponded with the higher quantitative yield (1–3.9 mg g^-1^) and higher level of TFA (4.8–9.9 mg g^-1^).

However, when MAR441^T^ was grown on a single nitrogen source with urea or (NH_4_)_2_SO_4_, this led to a low proportion of MUFAs and PUFAs in favour of SCFAs, due to an increase of n-13:0 (42.5% for urea and 51.1% for (NH_4_)_2_SO_4_ with corresponding decrease of n-16:1ω7, n-18:1ω7 and EPA, and less average chain length (14.79 for urea and 14.91 for (NH_4_)_2_SO_4_ in that the deficiency of carbon sources used for developing backbone.

Thus, ZB medium (ZB1) supplemented with L-proline and (NH_4_)_2_SO_4_ for growing MAR441^T^ cells led to a higher production of MUFAs and PUFAs with the proportion of 20.7% and 25.3% respectively due to the rise of n-16:1ω7 (20.4%) and EPA (17.6%), which was mirrored by the quantitative yield of TFA (115.2 mg g^-1^) and EPA (20.28 mg g^-1^), and higher ACL of 15.91.

### Effect of cerulenin treatment on the amount of biomass, lipids and EPA in MAR441^T^ cells

**Fig 4:**
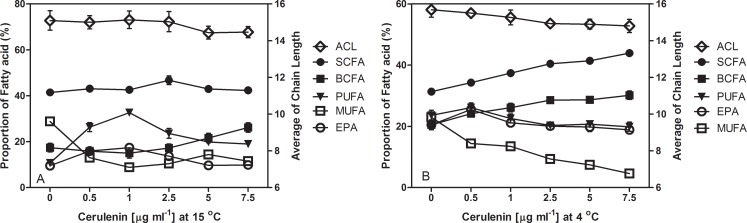
Change in average chain length (ACL, open diamonds) and relative proportion of whole cell FAs in strain MAR441^T^ grown in marine broth medium at 4°C **(A)** and 15°C **(B)** with various concentration of cerulenin. Straight chain fatty acids (SCFAs, filled circles), branched chain fatty acids (BCFAs, filled boxes), monounsaturated fatty acids (MUFAs, open boxes); polyunsaturated fatty acids (PUFAs, upside down triangles); eicosapentaenoic acid (EPA, open circles). The experiments were carried out in triplicate and values are means of three samples based on **S4 Table**.

Biomass production, lipid and EPA yields obtained from MAR441^T^ cells grown in the medium containing cerulenin up to 7.5 μg ml^-1^ at 4°C and 15°C are summarized in **Fig 4** and **S4 Table**. The cell biomass was around 3.2 g dry cells l^-1^ when cells were grown in the medium containing cerulenin at 0.5, 1, 2.5, 5 and 7.5 μg ml^-1^ respectively. The value was 20% increased as that of non-treated cells. The lipid yield (g l^-1^) was not changed by the concentration of cerulenin in the range from 0 to 7.5 μg ml^-1^. However, the biomass was slightly influenced by increasing the concentration of cerulenin to the medium up to 7.5 μg ml^-1^, indicating some effects of cerulenin on the growth of MAR441^T^ cells. The highest EPA yield at 81.5 μg ml^-1^ was obtained from cells treated with cerulenin at 0.5 μg ml^-1^ at 4°C. This yield was 53% increased over that obtained from non-treated cells. However, EPA production was 93% increased at 15°C when the cells were treated with 1 μg ml-1 cerulenin, whereas the percentage of PUFAs was 201% increased due to a marked increase of both n-18:2ω6t and n-18:3ω3 All these results clearly show that cerulenin treatment enhances EPA or PUFAs production as well as the short chain fatty acids, such as n-13:0 and n-15:0. mMost of the middle chain FAs, such as n-16:0, n-18:0, i-15:0, n-15:1ω6, n-16:1ω7, n-17:1ω8 and n-18:1ω7c acids were inhibited in MAR441^T^ cells when various concentration of cerulenin were included in the medium.

### Fatty acid production by NTG-mutant strains (A4 and A13)

**Fig 5:**
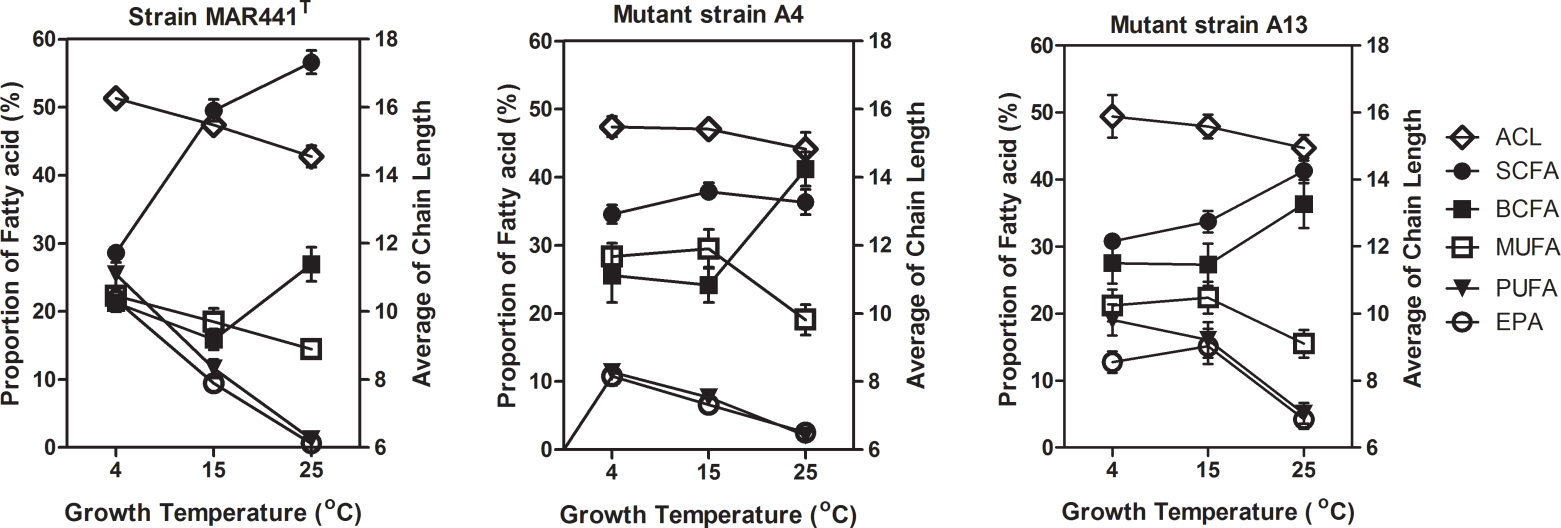
Change in average chain length (ACL, open diamonds) and relative proportion of whole cell FAs in strain MAR441^T^ and its NTG mutants (A4 and A13) grown at 4, 15 and 25°C. Straight chain fatty acids (SCFAs, filled circles), branched chain fatty acids (BCFAs, filled boxes), monounsaturated fatty acids (MUFAs, open boxes), polyunsaturated fatty acids (PUFAs, upside down triangles) and eicosapentaenoic acid (EPA, open circles). The experiments were carried out in triplicate and values are means of three samples based on **S5 Table**.

Two mutants A4 and A13 were morphologically different from the wild type strain MAR441^T^. Colonies of strain MAR441^T^ on marine agar plates were 2–4 mm in diameter, tan-pigmented, butyrous in consistency, circular and convex in shape with an smooth edge, and the central rough area was adherent to or embeds into the agar and was not easy to emulsify. Whereas, colonies of NTG-mutant strains A4 and A13 on marine agar plates were 3–5 mm in diameter, tan-pigmented, opaque, dull, with dentate margin or undulate edge, and the central rough area was attached to the agar loosely, and this was easy to be pushed/moved away by pipette tips (**S2A Fig**). Under scanning electron microscopy (SEM) cells of A4 and A13 were found to lack fimbriae after the NTG mutation (**S2B Fig**). The effect of growth temperature on the percentage composition of individual fatty acids in MAR441^T^ and its NTG-mutants (A4 and A13) grown at 4°C, 15°C and 25°C is shown in **Fig 5** and **S5 Table**. Comparing to the fatty acid compositions of strain MAR441^T^, the mutants were found with lower levels of SCFAs and higher percentage of BCFAs at 15°C and 25°C, and lower levels of EPA at 4°C. However, the percentage of EPA in mutant A13 was higher than that in wild type strain at 15°C, and the levels of EPA were decreased at 4°C and 25°C. By increasing temperatures from 4°C to 25°C, the values of ACL in these mutants decreased. The quantitative level of lipid content of the mutants were similar to that of MAR441^T^ at 4°C and 15°C, while increasing levels of EPA were found at 25°C. Mutant A13 could reach relatively higher level of EPA of 15.8 mg g^-1^ cells dry weight at 15°C (**S5 Table**).

## Discussion

### Environmental adaptation

The fatty acid composition of MAR441^T^ exhibited changes in response to growth temperature and sole carbon/nitrogen source, as has been reported for some *Shewanella* PUFAs producers [15,28], and non-PUFA producers, such as *Cobetia marina* [41]. Both the percentage and the quantitative level of EPA markedly changed at different growth temperatures, indicating that PUFA may play a critical role in the modulation of membrane fluidity [28,42]. The precise reason why these bacteria produce omega-3 fatty acids is still unclear, although many PUFA synthase genes responsible for EPA/DHA synthesis have been cloned and sequenced [43,44], and been successfully expressed in *E*. *coli* [5,11]. As growth temperature increased, MAR441^T^ also demonstrated a novel adaptive response by increasing the percentage of n-13:0 and i-15:0 with corresponding decrease of n-16:1ω7 and n-18:1ω7, which might be due to the role of fatty acid precursor selection in this bacterium as an adaptive response [45]. This finding is also supported by some other strains possessing similar fatty acid compositions, which share similar adaptive responses, such as *Shewanella gelidimarina* ACAM 456^T^, which exhibited an increased proportion of n-15:0 and a decrease of n-16:1ω7 and EPA with increasing culture temperature [28], *S*. *olleyana* ACEM 9^T^ showed an increase in the percentage of n-16:0 and i-15:0 with a corresponding decrease of n-16:1ω7, n-18:1ω7 and EPA when the growth temperature increased [20]. These observations indicate that PUFAs may play a key role in the homeostatic adaptation of cellular membrane viscosity and the modulation of membrane fluidity in these marine isolates.

Growth on the sole carbon sources L-proline, Tween 80, Tween 60 or Tween 40 demonstrated that in this bacterium the fatty acid composition can be manipulated by the provision of potential acyl chain primers [28]. There was a large increase in the observed percentage of EPA and lower levels of i-13:0 and i-15:0 in the L-proline cultures. While an observed increase of i-13:0 and to a lesser extent i-15:0 from L-alanine and L-leucine cultures that there is a preference for 4–5 cycles of chain elongation from the alanine- or leucine-derived primer molecule [46]. This is similar to *S*. *gelidimarina* ACAM 456^T^, which exhibited a similar change in fatty acid composition when it was grown in L-leucine medium [28]. The degree of acyl chain elongation may therefore be primer-specific. L-alanine and L-proline also increased the level of TFA, and therefore EPA, suggesting an increase in the level of total lipid resulted from growth on these substrates. In contrast, FA composition patterns were dominated by the n-18:1ω9 in Tween 80 cultures, while mainly n-18:0 in Tween 60 cultures and with rise of n-16:1ω7 when grown in Tween 40, which greatly decrease the percentage of PUFAs or polyunsaturation degree. These data are in agreement with the results obtained for EPA-producing *Shewanella* strain GA-22, the Tween 80-grown cells showed an increase of monounsaturated fatty acids, up to 78% in cellular lipids and an inhibition of the PUFA production [15]. These observations were a consequence of the nature of the added substrates, because Tween 80 contains oleic (n-18:1) acid, Tween 60 has stearic acids (n-18:0) and Tween 40 is composed of n-16:0. Although Tween 80, 60 and 40 usually serve as surfactants, they can also be used as carbon sources.

However, the strain preferred growing in the complex media, such as marine broth and ZoBell broth, from which the higher levels of biomass, lipid or TFA and EPA were produced. This corroborates previous reports that production of PUFA at low temperatures was enhanced more than two-fold and reached 5% of total fatty acids in the strain GA-22 cells grown on marine broth [15]. In our study, L-proline and (NH_4_)_2_SO_4_ were selected as the most suitable carbon and nitrogen sources used in combination for preparing ZB medium, which improved the production of biomass two-fold and EPA 1.5-fold.

MAR441^T^ required Na^+^ for growth and EPA synthesis and this is the case with other marine species such as *S*. *halifaxensis*, *S*. *sediminis*, *S*. *pealeana* and *S*. *woodyi*, which also preferred low temperatures for growth and are thus considered cold-adapted obligate species of *Shewanella* [47,48]. Studies on protein coding sequences from two obligate marine *S*. *halifaxensis* and *S*. *sediminis*, found that many genes coding Na^+^-dependent nutrient transporters were recruited to use the high Na^+^ content as an energy source. For example, many unique Na^+^-dependent nutrient symporters and Na^+^/nutrient symporters are used for transport of L-glutamine acid, L-proline, dicarboxylate and amino acids [49]. Based on genome annotations of *Shewanella* species, L-glutamine acid was predicted to be an essential precursor for biosynthesis of heme, nucleobase (purine, pyrimidine), peptidoglycan, aminosugar and fatty acids[50]. The requirement of Na^+^ as a motive force for transport of these essential growth substrates is consistent with the nature of strain MAR441^T^ being an obligate marine bacterium.

Cerulenin specifically blocks the activity of β-keto acyl thioester synthetase, which may account for the inhibition of fatty acid synthesis [51]. However, EPA production was greatly improved in MAR441^T^ treated by cerulenin, and middle-chain fatty acids were almost absent. These results suggest that cerulenin inhibited the *de novo* synthesis of middle-chain fatty acids, but not the synthesis of EPA and short-chain fatty acids. Therefore, strain MAR441^T^ may employ two fatty acid-biosynthetic systems and independently synthesize middle-chain fatty acids and LC-PUFAs using a common starting material as a FA precursor [24].

NTG mutagenesis is particularly useful for chemical mutagenesis of a variety of Gram-negative bacteria [52]. However, NTG mutagenesis has not previously been reported as a means for increasing PUFA production from environmental isolates of bacteria, although *S*. *putrefaciens* strain 2738 has been treated by NTG to get cold-sensitive EPA-requiring mutants at low temperatures [11]. Therefore, by treating MAR441^T^ with NTG at higher temperatures, we were able to obtain less temperature sensitive mutants with improved levels of lipid and EPA content when grown at higher temperatures.

### EPA yield

The yield of EPA from strain MAR441^T^ ranged from 2 to 20 mg g^-1^ (30 mg g^-1^ from the cerulenin-treated cultures) (cells dry weight) or 6 to 63 mg l^-1^ depending on culture conditions, and showed the highest quantitative yield of 11% TFA of dry cell weight and 20 mg l^-1^day^-1^ of EPA production in marine broth at 15°C for 1.5 days, values which compare favourably with literature reports for other EPA-producing bacteria. A *Shewanella putrefaciens*-like strain, SCRC-8132 produced 4–15 mg g^-1^ (cells dry weight) of EPA and 2% TFA of dry cell weight, although it was reported with high percentage of EPA (24–40%) [53,54]. Under various temperatures and utilizing different carbon sources, *Shewanella gelidimarina* ACAM 456^T^ produced 1–16 mg g^-1^ (cells dry weight) of EPA [28], whereas the *S*. *putrefaciens*-like strain SCRC-2738, is presently identified as *S*. *pneumatophori* SCRC-2738 [55], which produced 4–11 mg g^-1^ of EPA [56], the production was further enhanced to 17 mg g^-1^ [57].

Many wild-type strains of autophotrophic microalgae produce similar levels of EPA to the bacteria mentioned above, such as *P*. *tricornutum* and *Monodus subterraneus* [58]. Continuous culture of the heterotrophic diatom *Nitzschia laevis*, gave EPA productivities of 73 mg l^-1^ day^-1^ using a glucose feed [59]. In a high cell density system maximum cell dry weight and EPA yields were 22.1 g l^-1^ and 695 mg l^-1^, respectively, in a 14-day incubation [60]. While, the productivity of *P*. *tricornutum* by using batch culture was 25 mg l^-1^ day^-1^ [58], and improved up to 40 mg l^-1^ day^-1^ using a photobioreactor [61], *N*. *alba* ATCC 40775 gave an enhanced EPA production of 100–300 mg l^-1^ day^-1^ (40–50 g l^-1^ biomass) [62], *Thraustochytrid* strains were reported with an EPA productivity of 47 mg L^-1^ day^-1^ in a 50 l tubular photobioreactor [63], *Mortierella alpina* 1S-4 and *M*. *subterraneus* were reported with EPA production of 30 and 50 mg l^-1^ day^-1^ respectively [63], *M*. *alpine* was with high biomass production of 43 g l^-1^ and EPA productivity of 60 mg l^-1^ day^-1^ [64], and *Nannochloropsis oceanica* CY2 gave an EPA content and biomass concentration of 2.4% (per dry cell weight) and 1.5 g/l and productivity of 13.2 mg l^-1^ day^-1^ [65]. However, the majority of these algal species generally require strictly controlled growth conditions in terms of nutrients, light quantity and quality, oxygenation and carbon dioxide levels; these factors can result in considerable expense. In contrast, strain MAR441^T^ has a high EPA productivity of 20 mg l^-1^day^-1^ (2 g l^-1^ of biomass) when grown in marine broth at 15°C and could provide a cost effective and reliable source of PUFA. However, biomass yield per unit volume and culture time may also affect production economics. Nevertheless, the strain could be used as a feedstock for organisms, such as rotifers, as a way of introducing them into a marine food web for producing PUFA-rich oils by aquaculture.

### EPA in phospholipid and non-esterified fatty acids

The occurrence of non-esterified fatty acids (NEFA), or free fatty acids (FFA) in MAR441^T^ was identified using TLC and GC, accounting for 15% of the total lipid and containing 18.7% EPA. Phospholipids were found as the main component of total lipid (72% versus 28% for neutral lipid). The major phospholipid classes found were phosphatidyl ethanolamine (PE) and phosphatidyl glycerol (PG) as the main components (50% versus 40%), appreciable levels of EPA were detected from both fractions (9.5% versus 14.2% for PG) and 6.5% EPA in diphosphoglyceride (DPG), which is accounted for 5% of total lipid. The presence of non-esterified fatty acids within a PUFA-producing *Vibrio* sp. was previously reported to be 13.3% of total lipid, and EPA accounted for 13% of NEFA [66]. PE (with 5.5% of EPA) and PG (with 10.6% of EPA) accounted for 61% and 19% of phospholipids respectively in *Aeromonas* sp. 3010, and a high content of EPA and palmitoleic acid (19.7 and 50%, respectively) was found in the free fatty acid fraction [67]. For *Shewanella* sp. strain ACAM 456, PG contained a higher percentage of total PUFA (14% versus 9% for PG) and EPA (13% versus 9%), and NEFA accounted for 9% of total lipid and contained 22% EPA [28]. By employing FAB-MS-MS, acyl chains, such as i-13:0/13:0 and i-14:0/14:0 appeared to be associated with EPA in PE phospholipid species only, whereas the association of n-17:1 and acyl-18:0 chains with EPA was specific to PG in strain ACAM 456 [28]. Yazawa also reported the presence of NEFA in the PUFA producing strain SCRC-2738, with 5–10% of total EPA in the non-esterified form. This may be explained by the contribution of most EPA-producing bacterial genera possessing PG as their major phospholipid type, MUFAs and EPA were mostly concentrated in the PG component, while the proportion of branched-chain fatty acids was elevated in PE. Furthermore, the above mentioned five PUFA-producing bacteria all contain appreciable amounts of NEFA or FFA, of which EPA appears as a particular component of NEFA in PUFA metabolism. This remains an area for further investigation.

Genetically modified organisms (GMOs) as a source of ingredients have been used in our foods for over 20 years—the major argument for using them is that they offer "increased yield, drought tolerance, enhanced nutrition" and other consumer benefits [68]. However, the traditional approach of GMO risk assessment has been questioned [10], there is also an argument against GMOs due to the cause of health problems, environmental damage and violation of farmers and consumers rights [69]. Furthermore, consumers become more health conscious and knowledgeable about what they eat and where it comes from and if the food they eat contains GMOs, and they do try to avoid this [70]. Thus, GMOs as a source of dietary supplements is not recommended as a way to support better nourishment. Nevertheless, the number of companies which are publicly taking a stand against genetically modified foods is growing. For example, smaller natural foods companies are removing foods sourced from GMOs from their products. For example Popcorn Indiana's Fit Popcorn, Trader Joes' health and beauty products and Clif Bars [71]. Our approach to producing improved yields of omega-3 fatty acids using wild-type microorganisms, without using recombinant DNA techniques is an approach which is often preferred by an increasing number of ingredients manufacturers.

## Conclusions

This study reveals that temperature, medium composition, Na^+^ concentration, carbon and nitrogen sources, and the addition of cerulenin all play crucial roles in affecting bacterial growth and EPA accumulation in *Shewanella electrodiphila* MAR441^T^. The most efficient EPA production occurred when the strain was grown in modified Marine medium with L-proline and (NH_4_)_2_SO_4_ at a concentration of 1.50 g/l. The optimal EPA content (20–30 mg/g) and EPA productivity (20 mg/l/d) is among the highest levels yet reported, indicating the potential of using this system for the commercial production of EPA using this new wild-type marine bacterium.

## Supporting information

S1 Fig Lipid profiles from strain MAR441^T^ whole-cell lipid extracts separated on TLC plates. (DOC)

S2 Fig **(A)** Colonies of strain MAR441^T^ and its NTG mutants (A4 and A13) grown on marine agar plates at 15 ºC for 3 days; **(B)** scanning electron microscopy (Right, Bar 500 nm) of a negatively-stained cell of strain MAR441^T^ and its NTG mutants (A4 and A13).
(DOC)

S1 Table Fatty acid composition of strain MAR441^T^ during a time course of cell growth in marine broth medium at 15°C. (DOC)

S2 Table Fatty acid composition of strain MAR441^T^ grown on various concentrations of NaCl in ZB liquid medium at 15°C. (DOC)

S3 Table Fatty acid composition of strain MAR441^T^ grown on various sole carbon/nitrogen sources at 15°C. (DOC)

S4 Table Fatty acid composition of strain MAR441^T^ grown on various concentrations of cerulenin in marine broth medium at 4°C and 15°C. (DOC)

S5 Table Fatty acid composition of strain MAR441^T^ and its NTG mutants (A4 and A13) grown on marine broth at 15°C. (DOC)
