# Supplementary material for: Enhanced eicosapentaenoic acid production by a new deep-sea marine bacterium Shewanella electrodiphila MAR441T
Source: PLoS One. 2017 Nov 27;12(11):e0188081. doi: 10.1371/journal.pone.0188081 (PMC5703452; doi:10.1371/journal.pone.0188081)
Supplement: S1 Fig — (DOC) [file pone.0188081.s001.doc]

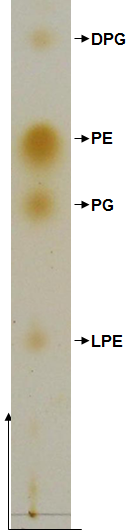


**S1 Fig** Lipid composition of whole-cell lipid extracts from strain MAR441^T^ separated on TLC plates. Major lipid components Diphosphatidylglycerol (DPG), Phosphatidyl ethanolamine (PE), Phosphatidyl glycerol (PG) and Lysophosphatidylethanolaminen (LPE) are indicated by arrows. TLC Solvent system used: CHCl_3_/MeOH/CH_3_COOH/H_2_O (85:15:10:3.5, v/v/v/v by vol.)
