# Supplementary material for: Enhanced eicosapentaenoic acid production by a new deep-sea marine bacterium Shewanella electrodiphila MAR441T
Source: PLoS One. 2017 Nov 27;12(11):e0188081. doi: 10.1371/journal.pone.0188081 (PMC5703452; doi:10.1371/journal.pone.0188081)
Supplement: S2 Fig — (A) Colonies of strain MAR441T and its NTG mutants (A4 and A13) grown on marine agar plates at 15 ºC for 3 days; (B) scanning electron microscopy (Right, Bar 500 nm) of a negatively-stained cell of strain MAR441T and its NTG mutants (A4 and A13). (DOC) [file pone.0188081.s002.doc]

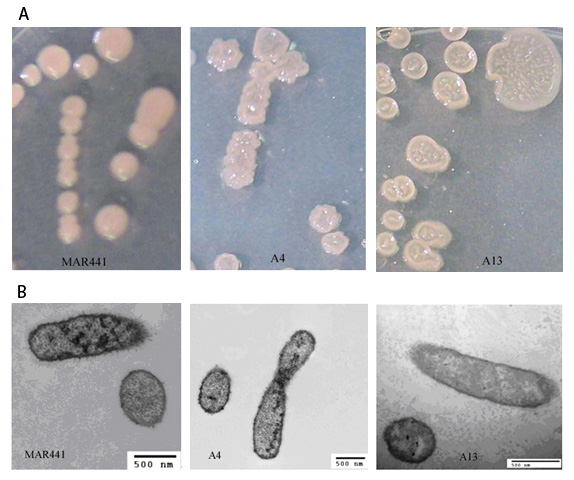


**S2 Fig (A)** Colonies of strain MAR441^T^ and its NTG mutants (A4 and A13) grown on marine agar plates at 15 ºC for 3 days; **(B)** scanning electron microscopy of negatively-stained cells of strain MAR441^T^ and its NTG mutants (A4 and A13) (Bar = 500 nm).
