# Supplementary material for: Enhanced eicosapentaenoic acid production by a new deep-sea marine bacterium Shewanella electrodiphila MAR441T
Source: PLoS One. 2017 Nov 27;12(11):e0188081. doi: 10.1371/journal.pone.0188081 (PMC5703452; doi:10.1371/journal.pone.0188081)
Supplement: S1 Table — (DOC) [file pone.0188081.s003.doc]

**S1** **Table** Fatty acid composition of strain MAR441^T^ during a time course of cell growth in marine broth medium at 15 °C

| Composition | Time period (hr) | | | | | | | | | | | | |
| --- | --- | --- | --- | --- | --- | --- | --- | --- | --- | --- | --- | --- | --- |
| Fatty acids | 12 | 18 | 24 | 36 | 42 | 48 | 60 | 66 | 72 | 84 | 96 | 108 | 132 |
| n-12:0 | 2.76 | 2.16 | 2.2 | 2.1 | 1.8 | 1.7 | 2.6 | 2.7 | 3.3 | 2.9 | 2.7 | 1 | 0.2 |
| n-13:0 | 42.09 | 37.6 | 31.92 | 22.92 | 20.6 | 21.1 | 22.5 | 24.1 | 23.5 | 29.2 | 33.5 | 40 | 63.1 |
| n-14:0 | 3.79 | 3.49 | 5.11 | 4.11 | 4.5 | 4.9 | 4.6 | 4.2 | 4.1 | 4.1 | 4.4 | 3.4 | 1.7 |
| n-15:0 | 1.71 | 1.77 | 2.12 | 2.25 | 2.6 | 2.1 | 2.4 | 2.4 | 2.7 | 1.3 | 0.3 | 0.5 | 0.6 |
| n-16:0 | 7.76 | 8.5 | 9.6 | 10.99 | 11.2 | 10.8 | 9.7 | 10.9 | 12.5 | 12.7 | 11.7 | 9.5 | 5.3 |
| n-17:0 | 0.33 | 0.31 | 0.87 | 0.56 | 0.6 | 0.3 | 0.5 | 0.6 | 0.5 | 0.3 | 0.1 | 0.59 | 0.6 |
| n-18:0 | 1.75 | 1.7 | 0.42 | 0.44 | 0.5 | 0.45 | 0.3 | 0.4 | 0.3 | 0.2 | 0.1 | 1 | 1.5 |
| **Σ SCFA** | **60.19** | **55.53** | **52.24** | **43.37** | **41.8** | **41.35** | **42.6** | **45.3** | **46.9** | **50.7** | **52.8** | **55.99** | **72.9** |
| i-13:0 | 6.34 | 5.12 | 6.1 | 7.23 | 8.23 | 8.5 | 9.1 | 8.2 | 7.1 | 6.5 | 5.1 | 5.3 | 2.7 |
| i-14:0 | 0.72 | 0.62 | 0.58 | 0.68 | 0.8 | 0.7 | 0.6 | 0.5 | 0.5 | 0.55 | 0.5 | 0.24 | 0.2 |
| ai-15:0 | 0.72 | 0.72 | 0.3 | 0.2 | 0.5 | 0.6 | 0.5 | 0.7 | 0.9 | 0.6 | 0.4 | 1.1 | 0.1 |
| i-15:0 | 5.35 | 4.7 | 6.7 | 10.29 | 12.2 | 11.6 | 11.7 | 12.3 | 11.8 | 9.8 | 8.9 | 4.93 | 4.9 |
| i-17:0 | 0.33 | 0.31 | 0.43 | 0.33 | 0.21 | 0.22 | 0.2 | 0.2 | 0.2 | 0.25 | 0.2 | 0.65 | 0.7 |
| **Σ BCFA** | **13.46** | **11.47** | **14.11** | **18.73** | **21.94** | **21.62** | **22.1** | **21.9** | **20.5** | **17.7** | **15.1** | **12.22** | **8.7** |
| n-15:1ω6 | 0.12 | 0.11 | 0.07 | 0.09 | 0.1 | 0.2 | 0.3 | 0.3 | 0.2 | 0.2 | 0.2 | 0.1 | 0.1 |
| n-16:1ω7 | 10.64 | 14.1 | 12.65 | 13.65 | 14.2 | 15 | 14.4 | 13.2 | 13.5 | 13.3 | 13.4 | 14.3 | 6.1 |
| n-17:1ω8 | 0.18 | 0.16 | 0.23 | 0.28 | 0.42 | 0.4 | 0.8 | 0.7 | 0.4 | 0.4 | 0.3 | 0.1 | 0.1 |
| n-18:1ω9c | 0.38 | 0.25 | 0.45 | 0.53 | 0.4 | 0.3 | 0.5 | 0.3 | 0.2 | 0.2 | 0.2 | 0.1 | 0.3 |
| n-18:1ω7c | 2.43 | 2.11 | 3.88 | 4.88 | 4.7 | 4.7 | 4.3 | 4.1 | 5.2 | 4.8 | 4.5 | 3.3 | 1.5 |
| n-20:1ω9 | 0.32 | 0.32 | 0.23 | 0.19 | 0.23 | 0.3 | 0.2 | 0.3 | 0.3 | 0.25 | 0.2 | 0.1 | 0.4 |
| **Σ MUFA** | **14.07** | **17.05** | **17.51** | **19.62** | **20.05** | **20.9** | **20.5** | **18.9** | **19.8** | **19.15** | **18.8** | **18** | **8.5** |
| n-18:2ω6t | 0.36 | 0.38 | 0.23 | 0.11 | 0.21 | 0.22 | 0.25 | 0.3 | 0.2 | 0.3 | 0.5 | 0.5 | 0.7 |
| n-18:3ω6t | - | - | 0.29 | 0.09 | 0.04 | 0.1 | 0.1 | 0.1 | 0.1 | 0.1 | - | - | 0.7 |
| n-18:3ω3 | 0.37 | 0.35 | 0.3 | 0.11 | 0.13 | 0.14 | 0.15 | 0.2 | 0.1 | 0.1 | 0.6 | 0.8 | 0.4 |
| n-18:4ω3 | 0.21 | 0.24 | 0.28 | 0.31 | 0.27 | 0.3 | 0.5 | 0.45 | 0.3 | 0.3 | 0.4 | 0.2 | - |
| n-20:2 | 0.3 | 0.32 | 0.18 | 0.08 | 0.08 | 0.05 | 0.1 | 0.15 | 0.1 | 0.2 | 0.3 | 0.7 | 0.7 |
| n-20:3ω6 | 0.1 | - | 0.14 | 0.04 | 0.05 | 0.07 | 0.1 | 0.12 | 0.1 | 0.3 | 0.5 | 0.3 | 0.4 |
| n-20:4ω6 | - | 0.11 | 0.23 | 0.2 | 0.14 | 0.15 | 0.18 | 0.2 | 0.25 | 0.2 | - | - | - |
| n-20:3ω3 | 0.69 | 0.71 | 0.66 | 0.06 | 0.08 | 0.08 | 0.1 | 0.11 | 0.2 | 0.3 | 0.4 | 0.5 | 0.5 |
| n-20:4ω3 | 0.23 | 0.26 | 0.34 | 0.59 | 0.49 | 0.53 | 0.63 | 0.53 | 0.5 | 0.4 | 0.1 | 0.1 | 0.1 |
| **n-20:5ω3** | **8.84** | **12** | **12.3** | **15.01** | **13.2** | **13.2** | **11.9** | **10.9** | **10** | **9.6** | **9.5** | **9.1** | **5.5** |
| n-22:2ω6 | - | - | - | 0.04 | 0.05 | 0.08 | 0.1 | 0.1 | 0.1 | 0.1 | - | - | - |
| n-22:4ω6 | 0.12 | 0.11 | 0.08 | 0.04 | 0.03 | 0.04 | 0.05 | 0.05 | 0.1 | 0.3 | 0.5 | 0.3 | 0.5 |
| n-22:5ω3 | 0.25 | 0.23 | 0.37 | 0.49 | 0.35 | 0.4 | 0.5 | 0.6 | 0.5 | 0.2 | 0.3 | 0.1 | 0.3 |
| **Σ PUFA** | **11.47** | **14.71** | **15.4** | **17.17** | **15.12** | **15.36** | **14.7** | **13.81** | **12.6** | **12.4** | **13.1** | **12.6** | **9.7** |
| Others | 0.81 | 0.74 | 0.74 | 1.11 | 1.09 | 0.77 | 0.14 | 0.09 | 0.25 | 0.8 | 0.21 | 0.89 | 0.0 |
| Total | 100 | 100 | 100 | 100 | 100 | 100 | 100 | 100 | 100 | 100 | 100 | 100 | 100.0 |
| ACL | 14.9 | 15.2 | 15.42 | 15.73 | 15.63 | 15.67 | 15.6 | 15.55 | 15.5 | 15.21 | 15.1 | 15.04 | 14.5 |
| EPA (mg g^-1^) | 6.9 | 9.6 | 11.1 | 15.48 | 14.1 | 14 | 13.4 | 12.1 | 11.2 | 10 | 8.5 | 7.7 | 4.7 |
| TFA (mg g^-1^) | 78.1 | 80.2 | 89.5 | 103.1 | 106.8 | 106.6 | 113 | 111 | 112 | 105 | 89.5 | 84.6 | 85.4 |
| Cells(g l^-1^) ^a^ | 0.6 | 0.8 | 1.4 | 1.96 | 2.42 | 2.66 | 2.7 | 2.68 | 2.7 | 2.6 | 2.5 | 2.2 | 2.2 |

^a^ Cellular dry weight; Values are means of three samples; ACL, average chain length); SCFA, straight chain fatty acids; BCFA, branched chain fatty acids; MUFA, monounsaturated fatty acids; PUFA, polyunsaturated fatty acids; TFA, total fatty acids; EPA, eicosapentaenoic acid (20:5ω3); and (–), not detectable.
