# Supplementary material for: Enhanced eicosapentaenoic acid production by a new deep-sea marine bacterium Shewanella electrodiphila MAR441T
Source: PLoS One. 2017 Nov 27;12(11):e0188081. doi: 10.1371/journal.pone.0188081 (PMC5703452; doi:10.1371/journal.pone.0188081)
Supplement: S2 Table — (DOC) [file pone.0188081.s004.doc]

**S2** **Table** Fatty acid composition of strain MAR441^T^ grown on various concentrations of NaCl in ZB liquid medium at 15 °C

| **Composition** | **NaCl (%)** | | | | | | | | | | |
| --- | --- | --- | --- | --- | --- | --- | --- | --- | --- | --- | --- |
| **Fatty acids** | **0** | **0.3** | **0.5** | **1.0** | **1.5** | **2.0** | **2.5** | **3.0** | **4.0** | **5.0** | **7.0** |
| n-12:0 | 0.7 | 3.0 | 1.9 | 2.8 | 1.7 | 2.3 | 1.8 | 2.0 | 2.8 | 3.5 | 1.7 |
| n-13:0 | 56.2 | 25.2 | 20.0 | 18.5 | 19.1 | 20.1 | 22.5 | 24.1 | 27.5 | 32.4 | 43.9 |
| n-14:0 | 0.9 | 4.4 | 3.9 | 4.4 | 2.9 | 3.3 | 3.0 | 2.9 | 3.0 | 3.1 | 0.9 |
| n-15:0 | 0.8 | 2.8 | 2.6 | 2.1 | 3.2 | 4.2 | 4.9 | 4.5 | 2.4 | 0.8 | 0.9 |
| n-16:0 | 4.2 | 8.4 | 12.1 | 12.4 | 11.3 | 9.1 | 8.3 | 8.1 | 4.7 | 2.2 | 6.7 |
| n-17:0 | 0.2 | 0.5 | 0.7 | 0.2 | 1.5 | 1.5 | 1.2 | 0.8 | 2.8 | 3.5 | 1.4 |
| n-18:0 | 1.5 | 2.1 | 1.3 | 2.0 | 2.2 | 1.5 | 1.2 | 1.3 | 2.2 | 2.8 | 2.0 |
| **Σ SCFA** | **64.5** | **46.4** | **42.4** | **42.4** | **41.9** | **42.0** | **42.9** | **43.6** | **45.4** | **48.3** | **57.5** |
| i-13:0 | 5.5 | 6.4 | 7.3 | 6.7 | 6.4 | 7.4 | 7.7 | 7.7 | 4.4 | 0.1 | 0.2 |
| i-14:0 | 0.6 | 0.5 | 0.7 | 0.3 | 0.5 | 0.5 | 0.3 | 0.4 | 0.2 | 0.2 | 0.2 |
| ai-15:0 | 0.2 | 0.5 | 0.7 | 0.4 | 0.5 | 0.6 | 0.5 | 0.4 | 0.3 | 0.2 | 0.2 |
| i-15:0 | 14.2 | 12.0 | 10.5 | 9.8 | 9.7 | 9.6 | 10.5 | 9.4 | 7.9 | 4.0 | 2.0 |
| i-17:0 | 1.4 | 0.1 | 0.2 | 0.1 | 0.2 | 0.1 | 0.1 | 0.1 | 0.1 | 0.2 | 0.3 |
| **Σ BCFA** | **21.8** | **19.5** | **19.3** | **17.3** | **17.2** | **18.2** | **19.1** | **18.1** | **12.9** | **4.7** | **2.9** |
| n-15:1ω6 | 1.8 | - | - | 0.5 | 0.5 | 0.6 | 0.8 | 0.9 | 6.9 | 8.1 | 9.3 |
| n-16:1ω7 | 5.3 | 12.2 | 15.2 | 16.2 | 16.2 | 15.7 | 14.8 | 16.7 | 18.0 | 19.2 | 22.4 |
| n-17:1ω8 | 0.2 | 2.5 | 1.5 | 2.5 | 3.9 | 5.3 | 4.8 | 3.0 | 0.5 | 0.2 | 0.3 |
| n-18:1ω9c | 0.5 | 0.2 | 0.6 | 0.5 | 0.5 | 0.2 | 0.5 | 0.4 | 0.3 | 0.2 | 0.2 |
| n-18:1ω7c | 1.9 | 7.6 | 5.1 | 5.6 | 5.5 | 5.3 | 5.9 | 6.1 | 6.4 | 7.2 | 3.3 |
| n-20:1ω9 | 0.5 | 0.1 | 0.1 | 0.2 | 0.2 | 0.1 | 0.1 | 0.2 | 0.3 | 0.3 | 0.4 |
| **Σ MUFA** | **10.1** | **22.6** | **22.5** | **25.5** | **26.9** | **27.2** | **26.9** | **27.3** | **32.4** | **35.2** | **35.9** |
| n-18:2ω6t | - | 0.2 | 0.3 | 0.8 | 0.8 | 0.6 | 0.7 | 0.5 | 1.0 | 1.5 | 0.1 |
| n-18:3ω6t | - | 0.2 | 0.3 | 0.3 | 0.3 | 0.3 | 0.1 | 0.2 | 0.2 | - | - |
| n-18:3ω3 | 1.0 | 0.3 | 0.3 | 0.8 | 0.9 | 1.0 | 0.4 | 0.6 | 0.6 | 1.8 | 0.1 |
| n-18:4ω3 | - | 0.5 | 0.6 | 0.7 | 0.7 | 0.6 | 1.7 | 1.3 | - | - | - |
| n-20:2 | 1.0 | 0.3 | 0.4 | 0.8 | 0.7 | 0.5 | 0.2 | 0.2 | 1.2 | 1.5 | 1.7 |
| n-20:4ω6 | - | 0.1 | 0.2 | 0.2 | 0.1 | 0.1 | 0.1 | 0.1 | 0.1 | - | - |
| n-20:3ω3 | 0.6 | 0.1 | 0.2 | 0.5 | 0.7 | 0.7 | 0.2 | 0.4 | 0.8 | 1.3 | 0.4 |
| n-20:4ω3 | - | 0.5 | 0.7 | 0.6 | 0.6 | 0.5 | 0.5 | 0.7 | - | - | - |
| **n-20:5ω3** | **0.2** | **8.6** | **11.9** | **8.6** | **7.8** | **6.8** | **6.2** | **5.8** | **3.2** | **1.6** | **0.4** |
| n-22:2ω6 | 0.3 | 0.1 | 0.1 | 0.4 | 0.4 | 0.4 | 0.1 | 0.3 | 0.8 | 0.8 | 0.3 |
| n-22:4ω6 | 0.3 | 0.1 | 0.1 | 0.5 | 0.3 | 0.2 | 0.2 | 0.2 | 0.9 | 0.6 | 0.3 |
| n-22:5ω3 | 0.2 | 0.3 | 0.4 | 0.4 | 0.5 | 0.3 | 0.3 | 0.3 | 0.4 | 0.4 | 0.3 |
| **Σ PUFA** | **3.6** | **11.3** | **15.4** | **14.6** | **13.9** | **12.0** | **10.7** | **10.6** | **9.2** | **9.5** | **3.7** |
| Others | 0.0 | 0.2 | 0.4 | 0.2 | 0.2 | 0.6 | 0.4 | 0.4 | 0.2 | 0.1 | 0.0 |
| Total | 100 | 100 | 100 | 100 | 100 | 100 | 100 | 100 | 100 | 100 | 100 |
| ACL | 14.27 | 15.32 | 15.58 | 15.63 | 15.69 | 15.42 | 15.32 | 15.28 | 15.26 | 14.93 | 14.77 |
| EPA (mg g^-1^) | 0.14 | 6.02 | 10.2 | 6.92 | 6.24 | 5.3 | 4.65 | 4.2 | 2.18 | 1.04 | 0.18 |
| TFA (mg g^-1^) | 68.1 | 70.1 | 85 | 80.5 | 80 | 78 | 75 | 70.5 | 68.2 | 65.3 | 45.3 |
| Cells(g l^-1^) ^a^ | 0.7 | 2 | 2.1 | 2.2 | 2.24 | 2 | 1.9 | 1.82 | 1.74 | 1.02 | 0.42 |

^a^ Cellular dry weight; Values are means of three samples; ACL, average chain length; SCFA, straight chain fatty acids; BCFA, branched chain fatty acids; MUFA, monounsaturated fatty acids; PUFA, polyunsaturated fatty acids; TFA, total fatty acids; EPA, eicosapentaenoic acid (20:5ω3); and (–), not detectable.
