# Supplementary material for: Enhanced eicosapentaenoic acid production by a new deep-sea marine bacterium Shewanella electrodiphila MAR441T
Source: PLoS One. 2017 Nov 27;12(11):e0188081. doi: 10.1371/journal.pone.0188081 (PMC5703452; doi:10.1371/journal.pone.0188081)
Supplement: S3 Table — (DOC) [file pone.0188081.s005.doc]

**S3 Table** Fatty acid composition of strain MAR441^T^ grown on various sole carbon/nitrogen sources at 15 °C

| **Composition** | **Medium** | | | | | | | | | | | | | | | |
| --- | --- | --- | --- | --- | --- | --- | --- | --- | --- | --- | --- | --- | --- | --- | --- | --- |
| **Fatty acids** | **OM** | **MB** | **ZB** | **NC** | **Alanine** | **Leucine** | **Proline** | **Serine** | **Pyruvate** | **Glucose** | **Glycerol** | **Tween 80** | **Tween 60** | **Tween 40** | **Urea** | **(NH_4_)_2_SO_4_** |
| n-12:0 | 2.3 | 2.1 | 1.6 | 1.9 | 2.9 | 1.2 | 2.2 | 2 | 2.4 | 2.4 | 2.8 | 0.3 | 2.7 | 3.3 | 1 | 0.5 |
| n-13:0 | 25 | 22.92 | 36.4 | 48.4 | 24.6 | 45.7 | 32.2 | 26.7 | 30.1 | 36.3 | 21 | 23.2 | 25.7 | 23.1 | 42.7 | 51.1 |
| n-14:0 | 3.3 | 4.11 | 4.1 | 2.8 | 4.2 | 2.4 | 3.3 | 3.6 | 4.8 | 3.2 | 3.5 | 2.6 | 3.6 | 1.7 | 2.2 | 0.8 |
| n-15:0 | 0.7 | 2.25 | 1.4 | 1.1 | 2.3 | - | 1.1 | 2.6 | 1.4 | 2 | 1.1 | 0.2 | 0.4 | 0.1 | 0 | 1.2 |
| n-16:0 | 13.8 | 10.99 | 9.2 | 8.3 | 10.7 | 7.2 | 9.7 | 11.6 | 11.8 | 9.8 | 11.1 | 8.1 | 10.8 | 7.2 | 8.2 | 6.3 |
| n-17:0 | 0.6 | 0.56 | 0.3 | 1.9 | 1.5 | 1.5 | 2.2 | 2.5 | 0.5 | 1.5 | 2.9 | 0.6 | 1 | 0.4 | 1.2 | 2.1 |
| n-18:0 | 1.9 | 0.44 | 2.4 | 3.1 | 1.9 | 2.5 | 2.3 | 4.2 | 2.1 | 2.8 | 1.2 | 3.1 | 33.3 | 15.4 | 3.2 | 2 |
| **Σ SCFA** | **47.6** | **43.37** | **55.4** | **67.5** | **48.1** | **60.5** | **53** | **53.2** | **53.1** | **58** | **43.6** | **38.1** | **77.5** | **51.2** | **58.5** | **64** |
| i-13:0 | 2.6 | 7.23 | 3.6 | 6.8 | 9.8 | 7.8 | 4.3 | 8.3 | 6.6 | 6.7 | 3.4 | 3 | 1.9 | 3.4 | 7.8 | 6.7 |
| i-14:0 | 0.9 | 0.33 | 0.4 | 0.2 | 0.3 | 0.5 | 0.2 | 0.1 | 0.4 | 0.6 | 0.4 | 0.6 | 0.2 | 0.15 | 0.2 | 0.25 |
| ai-15:0 | 0.2 | 0.68 | 0.3 | 0.1 | 0.1 | 0.15 | 0.2 | 0.3 | 0.2 | 0.5 | 0.1 | 0.3 | 0.3 | 0.2 | 0.23 | 0.3 |
| i-15:0 | 2.5 | 10.29 | 3 | 1.7 | 6 | 6.5 | 4.4 | 6 | 4.3 | 4 | 4.6 | 2.5 | 0.5 | 3.7 | 5.1 | 4 |
| i-17:0 | 0.2 | 0.54 | 0.4 | 0.44 | 0.15 | 0.24 | 0.21 | 0.5 | 0.38 | 0.49 | 0.42 | 1.2 | 0.11 | 0.15 | 0.36 | 0.13 |
| **Σ BCFA** | **6.4** | **19.07** | **7.7** | **9.24** | **16.35** | **15.19** | **9.31** | **15.2** | **11.88** | **12.29** | **8.92** | **7.6** | **3.01** | **7.6** | **13.69** | **11.38** |
| n-15:1ω6 | 0.1 | 0.09 | - | 0.2 | 0.26 | 0.3 | 0.1 | 0.5 | 0.4 | 0.3 | 0.1 | - | 0.5 | 0.7 | 0.2 | 0.1 |
| n-16:1ω7 | 20.4 | 13.65 | 14.1 | 10.5 | 14.6 | 9.1 | 11.3 | 11.4 | 13.6 | 12.1 | 28.8 | 10.6 | 5.4 | 24.7 | 8.9 | 6.2 |
| n-17:1ω8 | 0.1 | 0.28 | 0.2 | 2.2 | 2.3 | 2.2 | 1.6 | 1.2 | 0.6 | 1.2 | 0.7 | 1.3 | 2 | 1.5 | 2.1 | 3 |
| n-18:1ω9c | 0.3 | 0.53 | 0.3 | 0.1 | 0.3 | 0.2 | 0.1 | 0.1 | 0.2 | 0.3 | 0.2 | 35.3 | 0.4 | 0.6 | 0.2 | 0.5 |
| n-18:1ω7c | 4 | 4.88 | 2.8 | 4.4 | 3.3 | 2.1 | 7.1 | 4.8 | 6.1 | 3.6 | 4.4 | 1.5 | 3.8 | 4.5 | 6.4 | 2.5 |
| n-20:1ω9 | 0.4 | 0.19 | 0.1 | 0.3 | 0.2 | 0.6 | 0.1 | 0.2 | 0.4 | 0.2 | 0.5 | 0.1 | 0.1 | - | 0.2 | 0.4 |
| **Σ MUFA** | **25.3** | **19.62** | **17.5** | **17.7** | **20.96** | **14.5** | **20.3** | **18.2** | **21.3** | **17.7** | **34.7** | **48.8** | **12.2** | **32** | **18** | **12.7** |
| n-18:2ω6t | 0.5 | 1.16 | 0.7 | 0.27 | 0.15 | 0.25 | 0.11 | 0.13 | 0.14 | 0.15 | 0.2 | 0.1 | 0.35 | 0.21 | 0.54 | 0.3 |
| n-18:3ω6t | 0.5 | 0.09 | 0.7 | 0.2 | 0.25 | 0.38 | 0.31 | 0.27 | 0.3 | 0.5 | 0.45 | 0.35 | 0.24 | - | 0.21 | - |
| n-18:3ω3 | 0.2 | 0.11 | 0.1 | 0.2 | 0.12 | 0.28 | 0.08 | 0.21 | 0.15 | 0.25 | 0.4 | 0.15 | 0.3 | 0.6 | 0.53 | 0.7 |
| n-18:4ω3 | 0.4 | 0.31 | 0.8 | 0.15 | - | 0.17 | 0.04 | 0.15 | 0.2 | 0.1 | 0.1 | 0.2 | 0.5 | 0.34 | 0.22 | 0.4 |
| n-20:2 | 0.5 | 0.08 | 0.5 | 0.3 | 0.25 | 0.33 | 0.2 | 0.14 | 0.15 | 0.48 | 0.2 | 0.1 | - | 0.4 | 0.1 | - |
| n-20:3ω3 | 0.3 | 0.06 | 0.1 | 0.29 | 0.41 | 0.26 | 0.06 | 0.28 | 0.08 | 0.1 | 0.1 | 0.2 | 0.4 | 0.7 | 0.5 | 0.5 |
| n-20:4ω3 | 0.3 | 0.59 | 0.2 | 0.35 | 0.26 | 0.54 | 0.59 | 0.49 | 0.53 | 0.63 | 0.53 | 0.5 | 0.1 | 0.3 | 0.1 | 0.1 |
| **n-20:5ω3** | **17.6** | **15.01** | **13.8** | **3.4** | **12.8** | **6.4** | **15.6** | **11** | **11.4** | **9.4** | **10.2** | **3.2** | **4.5** | **5.6** | **6.2** | **8.5** |
| n-22:4ω6 | 0.1 | 0.04 | 2.2 | 0.3 | 0.2 | 1.1 | 0.2 | 0.4 | 0.35 | 0.2 | 0.1 | 0.6 | 0.75 | 0.8 | 1.3 | 1.2 |
| n-22:5ω3 | 0.3 | 0.49 | 0.3 | 0.1 | 0.15 | 0.1 | 0.2 | 0.33 | 0.42 | 0.2 | 0.5 | 0.1 | 0.15 | 0.25 | 0.11 | 0.25 |
| **Σ PUFA** | **20.7** | **17.94** | **19.4** | **5.56** | **14.59** | **9.81** | **17.39** | **13.4** | **13.72** | **12.01** | **12.78** | **5.5** | **7.29** | **9.2** | **9.81** | **11.9** |
| Total | 100 | 100 | 100 | 100 | 100 | 100 | 100 | 100 | 100 | 100 | 100 | 100 | 100 | 100 | 100 | 100 |
| ACL | 15.91 | 15.57 | 15.49 | 14.53 | 15.39 | 14.73 | 15.58 | 15.38 | 15.32 | 15.03 | 15.62 | 16.16 | 16.03 | 15.79 | 14.91 | 14.79 |
| **EPA (mg g^-1^)** | **20.28** | **15.48** | **14.2** | **1.9** | **12.57** | **5.68** | **15.23** | **11.13** | **11.00** | **9.94** | **10.66** | **7.26** | **4.06** | **5.18** | **5.90** | **8.19** |
| TFA (mg g**^-1^**) | 115.2 | 103.1 | 102.5 | 55.3 | 98.2 | 88.7 | 97.6 | 101.2 | 96.5 | 105.7 | 104.5 | 88.5 | 90.2 | 92.5 | 95.1 | 96.3 |
| Cells(g l**^-1^**) ^a^ | 3.1 | 2.66 | 2.6 | 0.75 | 1.95 | 2.1 | 2 | 1.85 | 2 | 2.3 | 2.2 | 1.65 | 1.7 | 1.5 | 1.8 | 1.85 |

^a^ Cellular dry weight; Values are means of three samples; ACL, average chain length; SCFA, straight chain fatty acids; BCFA,

branched chain fatty acids; MUFA, monounsaturated fatty acids; PUFA, polyunsaturated fatty acids; TFA, total fatty acids;

EPA, eicosapentaenoic acid (20:5ω3); and (–), not detectable. MB (marine broth); ZB (Zobell’s broth);

OM (Optimal medium, with 0.5% Proline and (NH_4_)_2_SO_4_) in NC; NC (negative control medium);
