# Supplementary material for: Enhanced eicosapentaenoic acid production by a new deep-sea marine bacterium Shewanella electrodiphila MAR441T
Source: PLoS One. 2017 Nov 27;12(11):e0188081. doi: 10.1371/journal.pone.0188081 (PMC5703452; doi:10.1371/journal.pone.0188081)
Supplement: S4 Table — (DOC) [file pone.0188081.s006.doc]

**S4** **Table** Fatty acid composition of strain MAR441^T^ grown on various concentrations of cerulenin in marine broth medium at 4°C and 15°C

| Composition | Cerulenin (µg ml^-1^) 15 °C | | | |  |  | Cerulenin (µg ml^-1^) 4 °C | | | |  |  |
| --- | --- | --- | --- | --- | --- | --- | --- | --- | --- | --- | --- | --- |
| Fatty acids | 0 | 0.5 | 1 | 2.5 | 5 | 7.5 | 0 | 0.5 | 1 | 2.5 | 5 | 7.5 |
| n-12:0 | 3.6 | 5.4 | 5.7 | 8.1 | 7.5 | 6.6 | 2.3 | 2.1 | 2.5 | 2.7 | 3.1 | 4.5 |
| n-13:0 | 16.8 | 17.7 | 19.4 | 20.9 | 17.3 | 16.3 | 13.5 | 14.5 | 17.1 | 18.5 | 19.7 | 21.5 |
| n-14:0 | 2.9 | 4.1 | 4.2 | 5.8 | 4.1 | 4.5 | 3.9 | 3.7 | 4.1 | 4.2 | 4.7 | 4.8 |
| n-15:0 | 4.7 | 10.8 | 11.6 | 12.1 | 13.5 | 14.3 | 4.5 | 7.3 | 8.5 | 11.5 | 12.3 | 11.1 |
| n-16:0 | 11.5 | 5.3 | 1.2 | 0.6 | 0.5 | 0.5 | 6.4 | 5.6 | 5.1 | 3.4 | 1.7 | 2.1 |
| n-18:0 | 2.4 | 0.0 | 0.0 | 0.0 | 0.0 | 0.0 | 0.5 | 0.8 | 0.1 | - | - | - |
| Σ SCFA | 41.9 | 43.3 | 42.1 | 47.5 | 42.9 | 42.2 | 31.1 | 34.0 | 37.4 | 40.3 | 41.5 | 44.0 |
| i-13:0 | 5.9 | 8.7 | 8.0 | 12.0 | 14.3 | 15.3 | 12.7 | 17.8 | 20.6 | 22.9 | 23.8 | 25.8 |
| i-15:0 | 11.2 | 7.7 | 6.8 | 5.5 | 7.5 | 10.8 | 7.7 | 6.2 | 5.5 | 5.3 | 5.1 | 4.5 |
| Σ BCFA | 17.1 | 16.4 | 14.8 | 17.5 | 21.8 | 26.1 | 20.4 | 24.0 | 26.1 | 28.2 | 28.9 | 30.3 |
| n-15:1ω6 | 0.9 | 0.2 | 0.6 | 0.2 | 1.7 | 1.6 | 0.1 | 0.6 | 0.6 | 0.3 | 0.2 | 0.3 |
| n-16:1ω7 | 18.2 | 10.9 | 6.3 | 8.1 | 10.8 | 8.5 | 16.9 | 13.4 | 12.6 | 9.1 | 7.2 | 4.3 |
| n-17:1ω8 | 3.8 | 0.1 | 0.1 | 0.0 | 0.4 | 0.0 | 0.2 | 0.1 | - | - | - | - |
| n-18:1ω7c | 5.9 | 1.8 | 2.0 | 1.8 | 1.5 | 1.3 | 6.0 | - | - | - | - | - |
| Σ MUFA | 28.8 | 13.0 | 9.0 | 10.2 | 14.4 | 11.4 | 23.2 | 14.1 | 13.2 | 9.4 | 7.5 | 4.6 |
| n-18:2ω6t | 0.5 | 6.3 | 9.1 | 7.6 | 7.2 | 7.1 | 2.8 | 0.5 | 0.4 | 0.4 | 0.5 | 0.4 |
| n-18:3ω3 | 0.6 | 3.8 | 5.4 | 2.9 | 2.7 | 2.1 | 0.3 | 0.2 | 0.7 | 0.7 | 0.8 | 0.7 |
| n-20:5ω3 | 9.5 | 16.1 | 18.3 | 13.1 | 9.5 | 9.6 | 20.5 | 25.5 | 20.9 | 19.5 | 19.2 | 18.6 |
| Σ PUFA | 10.6 | 26.2 | 32.8 | 23.7 | 19.4 | 18.8 | 23.5 | 26.2 | 22.0 | 20.6 | 20.5 | 19.6 |
| Others | 1.6 | 1.1 | 1.3 | 1.2 | 1.5 | 1.5 | 1.8 | 1.7 | 1.3 | 1.5 | 1.7 | 1.5 |
| Total | 100.0 | 100.0 | 100.0 | 100.0 | 100.0 | 100.0 | 100.0 | 100.0 | 100.0 | 100.0 | 100.0 | 100.0 |
| UFAs/SFAs | 0.9 | 0.9 | 1.0 | 0.7 | 0.8 | 0.7 | 1.5 | 1.2 | 0.9 | 0.7 | 0.7 | 0.5 |
| ACL | 15.3 | 15.4 | 15.5 | 15.0 | 14.8 | 14.8 | 15.65 | 15.5 | 15.2 | 15.0 | 14.8 | 14.7 |
| EPA (mg g^-1^) | 9.7 | 16.7 | 18.7 | 13.3 | 9.7 | 9.8 | 23.6 | 30.2 | 25.5 | 23.5 | 22.9 | 21.9 |
| TFA (mg g^-1^) | 102.5 | 103.5 | 102.0 | 101.5 | 102.1 | 102.2 | 115.2 | 118.6 | 122.0 | 120.5 | 119.1 | 118.0 |
| EPA(mg l^-1^) | 25.2 | 54.3 | 59.8 | 42.6 | 30.9 | 30.6 | 53.1 | 81.5 | 75.2 | 65.8 | 64.1 | 59.1 |
| Cells(g l^-1^) ^a^ | 2.6 | 3.25 | 3.2 | 3.2 | 3.19 | 3.12 | 2.25 | 2.7 | 2.95 | 2.8 | 2.8 | 2.7 |

^a^ Cellular dry weight; Values are means of three samples; ACL, average chain length; SCFA, straight chain fatty acids; BCFA, branched chain fatty acids; MUFA, monounsaturated fatty acids; PUFA, polyunsaturated fatty acids;

TFA, total fatty acids; EPA, eicosapentaenoic acid (20:5ω3); and (–), not detectable.
