# Supplementary material for: Enhanced eicosapentaenoic acid production by a new deep-sea marine bacterium Shewanella electrodiphila MAR441T
Source: PLoS One. 2017 Nov 27;12(11):e0188081. doi: 10.1371/journal.pone.0188081 (PMC5703452; doi:10.1371/journal.pone.0188081)
Supplement: S5 Table — (DOC) [file pone.0188081.s007.doc]

**S5** **Table** Fatty acid composition of strain MAR441^T^ and its NTG mutants (A4 and A13) grown on marine broth at 15 °C

|  | Temperatures | | | | | | | |  |
| --- | --- | --- | --- | --- | --- | --- | --- | --- | --- |
| Strain | MAR441^T^ |  |  | A4 |  |  | A13 |  |  |
| Fatty acids | 4°C | 15°C | 25°C | 4°C | 15°C | 25°C | 4°C | 15°C | 25°C |
| n-12:0 | 1.3^b^ | 2.2 | 0.3 | 4 | 4.5 | 5.5 | 3.8 | 3.6 | 4.6 |
| n-13:0 | 13.5 | 23.6 | 32.1 | 8.3 | 7.2 | 9.5 | 7.2 | 8.2 | 9.7 |
| n-14:0 | 3.9 | 2.9 | 2.7 | 3.7 | 4.4 | 6.1 | 4.5 | 4.7 | 6.5 |
| n-15:0 | 3.5 | 4.5 | 1.9 | 0.5 | 0.8 | 0.7 | 0.4 | 1.9 | 0.5 |
| n-16:0 | 5.3 | 14.5 | 12.4 | 14.1 | 17.9 | 9.3 | 12.4 | 12.3 | 16.1 |
| n-17:0 | 0.3 | 0.8 | 5.7 | 1 | 0.8 | 4.8 | 0.9 | 0.8 | 2.2 |
| n-18:0 | 0.5 | 1.3 | 1.0 | 3 | 2.2 | 0.3 | 2.1 | 1.7 | 2.3 |
| Σ SCFA | 28.3 | 49.7 | 56.0 | 34.6 | 37.8 | 36.2 | 31.3 | 33.2 | 41.9 |
| i-13:0 | 11.6 | 4.9 | 11.2 | 16.5 | 10.1 | 12.2 | 14.2 | 9.1 | 13.1 |
| i-14:0 | 0.7 | 0.4 | 0.2 | 1.5 | 0.5 | 0.9 | - | - | 0.3 |
| ai-15:0 | 1.2 | 0.4 | 0.8 | - | - | - | 0.4 | 0.7 | 0.4 |
| i-15:0 | 7.2 | 10.2 | 14.2 | 6.6 | 13.3 | 27.6 | 11.1 | 16.6 | 22.7 |
| i-17:0 | 0.5 | 0.2 | 0.2 | 0.4 | 0.3 | 0.5 | 1.7 | 0.5 | 0.4 |
| Σ BCFA | 21.2 | 16.2 | 26.6 | 25 | 24.2 | 41.2 | 27.4 | 26.9 | 36.9 |
| n-16:1ω7 | 16.9 | 11.2 | 9.3 | 18.4 | 20.2 | 13.1 | 15.2 | 17.9 | 12.1 |
| n-17:1ω8 | 0.2 | 2.8 | 4.0 | 3.3 | 2.6 | 2.5 | 2.1 | 2.1 | 2.1 |
| n-18:1ω7c | 5.2 | 4.2 | 1.1 | 6.3 | 6.4 | 3.5 | 4.2 | 2.8 | 1.2 |
| Σ MUFA | 22.3 | 18.3 | 14.5 | 28 | 29.2 | 19.1 | 21.5 | 22.8 | 15.4 |
| n-18:2ω6t | 2.8 | 1.5 | 0.3 | 0.3 | 0.4 | - | 0.3 | 0.5 | 0.5 |
| n-18:3ω3 | 0.3 | 0.6 | 0.2 | 0.5 | 0.3 | - | 0.3 | 0.3 | - |
| n-20:3ω3 | 0.8 | 0.3 | 0.1 | - | - | - | 5.7 | - | - |
| n-20:5ω3 | 21.4 | 9.5 | 0.6 | 10.7 | 6.8 | 2.6 | 12.8 | 15.3 | 4.5 |
| Σ PUFA | 25.3 | 11.9 | 1.2 | 11.5 | 7.5 | 2.6 | 19.1 | 16.1 | 5 |
| others | 2.9 | 3.9 | 1.9 | 0.9 | 1.3 | 0.9 | 0.7 | 1 | 0.8 |
| Total | 100 | 100 | 100 | 100 | 100 | 100 | 100 | 100 | 100 |
| ACL | 16.29 | 15.52 | 14.67 | 15.45 | 15.35 | 14.82 | 15.92 | 15.64 | 14.94 |
| EPA (mg g^-1^) | 24.7 | 9.7 | 0.6 | 10.7 | 6.8 | 2.4 | 12.8 | 15.8 | 4.3 |
| TFA (mg g^-1^) | 115.2 | 102.5 | 96.5 | 112.7 | 100.6 | 91.2 | 110.4 | 103.7 | 95.6 |
| Cells(g l^-1^) ^a^ | 2.25 | 2.6 | 0.6 | 2.3 | 2.6 | 0.62 | 2.35 | 2.66 | 0.6 |

^a^ Cellular dry weight; ^b^ Values are means of three samples; ACL, average chain length; SCFA, straight chain fatty acids; BCFA, branched chain fatty acids; MUFA, monounsaturated fatty acids; PUFA, polyunsaturated fatty acids; TFA, total fatty acids; EPA, eicosapentaenoic acid (20:5ω3); and (–), not detectable.
